# Pair-rule-like transcription patterns during neural tube closure in a proto-vertebrate

**DOI:** 10.1242/dev.205064

**Published:** 2025-12-15

**Authors:** Gabrielle Östlund-Sholars, Laurence A. Lemaire, Michael S. Levine

**Affiliations:** ^1^ Department of Molecular Biology, Princeton University, Princeton, NJ 08544, USA; ^2^ Lewis-Sigler Institute for Integrative Genomics, Princeton University, Princeton, NJ 08544, USA; ^3^ Department of Biology, Saint Louis University, St. Louis, MO 63108, USA

**Keywords:** Neurulation, Gene regulation, Patterning, Lmx1, Cell-cycle, *Ciona*

## Abstract

Neural tube closure (NTC) is a conserved morphogenetic process in chordates in which the neural plate folds and fuses to form a closed neural tube. While the mechanical forces and signaling pathways governing NTC have been characterized in vertebrates, the transcriptional programs coordinating these behaviors remain less understood. Here, we identify a transcriptional circuit involving *Lmx1*, *Cdkn1b* and *Msx* that regulates dorsal midline dynamics during NTC in the tunicate *Ciona*. High-resolution HCR *in situ* hybridization reveals that *Lmx1* expression is dynamically enriched at the zippering point and advances in a posterior-to-anterior transcription wave, while *Msx* is downregulated in the same region, marking a transition from early neural patterning to morphogenesis. As closure progresses, *Lmx1* and *Cdkn1b* exhibit complementary, alternating expression at the dorsal midline, resembling a pair-rule-like pattern. Misexpression experiments show that *Lmx1* promotes proliferation and autoregulates, whereas *Cdkn1b* limits proliferation and impedes closure. Single-cell RNA-seq datasets reveal transcriptionally distinct dorsal neural populations enriched for *Lmx1* or *Cdkn1b*. This transcriptional switch coordinates proliferation and fusion during NTC, suggesting a general strategy for regulating epithelial remodeling in animal embryos.

## Teaser

**Summary:** This study reports a striking pair-rule-like transcriptional logic associated with proliferation and morphogenesis during anterior neural tube closure in *Ciona*.

## INTRODUCTION

Neural tube closure (NTC) is a fundamental morphogenetic process in chordate development, during which the neural plate folds and fuses to form a hollow tube that gives rise to the central nervous system (Moon and Xiong, 2022; Nikolopoulou et al., 2017). In tunicates, this process proceeds through coordinated steps of neural plate bending, elevation of the neural folds, and a unidirectional posterior-to-anterior ‘zippering’ of the neural folds across the dorsal midline (Hashimoto and Munro, 2019; Hashimoto et al., 2015), similar to the multiple closure points of vertebrates. This process relies on the coordinated integration of mechanical forces and signaling cues from both neural and surrounding tissues to ensure timely and ordered closure (Nikolopoulou et al., 2017). Disruptions in NTC can lead to neural tube defects (NTDs), which are among the most prevalent congenital malformations in humans (Kancherla et al., 2023; Avagliano et al., 2019; Greene and Copp, 2014), including conditions such as spina bifida and anencephaly.

Recent studies highlight the importance of spatiotemporal control of cell proliferation during NTC (Ogura et al., 2011). In vertebrates, spatial differences in the cell-cycle state influence epithelial stiffness, tension generation and tissue fluidity, ultimately shaping morphogenetic outcomes (Bocanegra-Moreno et al., 2023). For example, decreased proliferation can impair convergent extension, while excessive proliferation can cause overcrowding and mechanical jamming (Bocanegra-Moreno et al., 2023; Kim et al., 2021). However, how these proliferative dynamics interface with patterning and cell fate specification remains difficult to resolve in vertebrates, in part because changes in proliferation inherently alter total cell number. The ascidian *Ciona* provides a simple system for studying this process due to its invariant cell lineages and small cell numbers.

There are striking parallels between NTC in *Ciona* and vertebrates (Sasakura et al., 2012; Dahlberg et al., 2009). During neurulation, mitotic divisions propagate in a posterior-to-anterior wave across the epidermis, synchronized with neural fold fusion. This mitotic wave is transcriptionally patterned via *GATAb* and *AP-2* acting on the cell-cycle regulator *cdc25* (Ogura and Sasakura, 2016). This mechanism establishes cell-cycle compensation in the epidermis to ensure seamless fusion at the neural/epidermal (Ne/Epi) boundary. Cell-cycle compensation refers to the local acceleration or delay of cell divisions to equalize cell numbers across regions undergoing morphogenetic strain. Dysregulation of compensation prevents the progression of neural fold fusion (Ogura et al., 2011). These findings highlight how transcriptional regulation of cell-cycle dynamics facilitates coordinated epithelial remodeling (Ogura and Sasakura, 2016). In this context, cell-cycle inhibitors such as *Cdkn1b* (a cyclin-dependent kinase inhibitor and *Ciona* homolog of CDKN1B/p27) emerge as important modulators of local proliferation (Treen et al., 2023b; Kobayashi et al., 2022). Although compensatory cell-cycle regulation has been studied in the overlying epidermis, it has not been characterized in the underlying neural cells that undergo neural tube zippering (Ogura and Sasakura, 2016).

In *Ciona*, zippering is driven by patterned actomyosin contractility and junctional exchange in dorsal neural tube cells. This behavior depends on local cytoskeletal asymmetry and differential cell-cell adhesion (Hashimoto and Munro, 2019; Hashimoto et al., 2015). These findings link mechanical force generation to regional cell behaviors, raising the possibility that transcriptional patterning directly coordinates morphogenetic behaviors during neurulation.

Transcription factors that simultaneously influence proliferation and cell fate likely contribute to coordinating proliferation and cell behaviors during NTC (Kim et al., 2022; Harris and Juriloff, 2010, 2007). LIM-homeodomain transcription factors, including LMX1A and LMX1B, are expressed in the dorsal midline of the vertebrate neural tube and are essential for roof plate specification and therefore dorsoventral patterning (Mishima et al., 2009; Chizhikov and Millen, 2004). These proteins regulate the expression of morphogens such as Wnt and BMP and are necessary for the organization of dorsal midline cells and proper neural tube morphology (Lehr et al., 2025; Yan et al., 2011; Mishima et al., 2009; Riddle et al., 1995). In mice, spontaneous mutations in *Lmx1a* result in the *Dreher* phenotype, characterized by roof plate loss and cerebellar hypoplasia, underscoring its role in proper dorsal central nervous system patterning (Millonig et al., 2000). At later stages, *Lmx1a* and *Lmx1b* promote the proliferation of midbrain dopaminergic progenitors (Yan et al., 2011). In both vertebrates and *Ciona*, Msx (muscle segment homeobox) genes mark the dorsal neural plate border and have been implicated in early neural patterning (Ramos and Robert, 2005; Imai et al., 2004), suggesting that multiple transcriptional programs contribute to dorsal midline organization.

Here, we employ single-cell methods to investigate the role of *Lmx1* in NTC in *Ciona*. We show that *Lmx1* is dynamically expressed in a posterior-to-anterior transcription wave at the advancing edge of closure, where it promotes cell proliferation and positively autoregulates its own transcription. Notably, we find that *Lmx1* might also activate its own expression in neighboring cells, raising the possibility of non-cell-autonomous expansion of the proliferative domain. *Lmx1* and *Msx* (also known as *Msxb*) are co-expressed in neural cells at the dorsal midline in early neurulation. As closure progresses, *Lmx1* becomes enriched at the zippering point while *Msx* is selectively downregulated, producing a locally exclusive pattern. This transition marks a shift from *Msx*-driven neural patterning to *Lmx1*-associated morphogenesis. As closure advances, *Lmx1* and *Cdkn1b* adopt a complementary, alternating expression pattern along the neural folds. Functional perturbations reveal that both excessive and insufficient proliferation of future midline cells disrupt closure, underscoring the importance of spatially patterned proliferation. High-resolution imaging and single-cell RNA-seq confirm transcriptionally distinct dorsal neural populations. Together, these findings suggest a pair-rule-like transcriptional logic involved in proliferation and morphogenesis, whereby alternating transcriptional states regulate epithelial remodeling. Our work identifies a previously uncharacterized role for *Lmx1* in morphogenesis, positioning it as a key node linking patterning, proliferation and cell behavior in a chordate model of NTC.

## RESULTS

### *Lmx1* expression advances in a posterior-to-anterior transcription wave during NTC

The *Ciona* genome contains two copies of *Lmx1* (*Lmx1* and *Lmx1-related*), both orthologous to the human paralog *LMX1B* (José-Edwards et al., 2011). Similar to their vertebrate counterparts, both *Ciona Lmx1* genes encode proteins with two tandem N-terminal LIM domains (each with a double zinc-finger motif) and a C-terminal DNA-binding homeodomain (German et al., 1992). While *Lmx1-related* is expressed ventrally in the developing notochord (Negrón-Piñeiro et al., 2024), *Lmx1* is expressed dorsally in the cells of the roof plate that zipper during NTC (Cao et al., 2019; Imai et al., 2004). In vertebrates, *Lmx1a/b* are essential for dorsal midline patterning and roof plate formation (Millonig et al., 2000; Riddle et al., 1995).

**Fig. 1.**
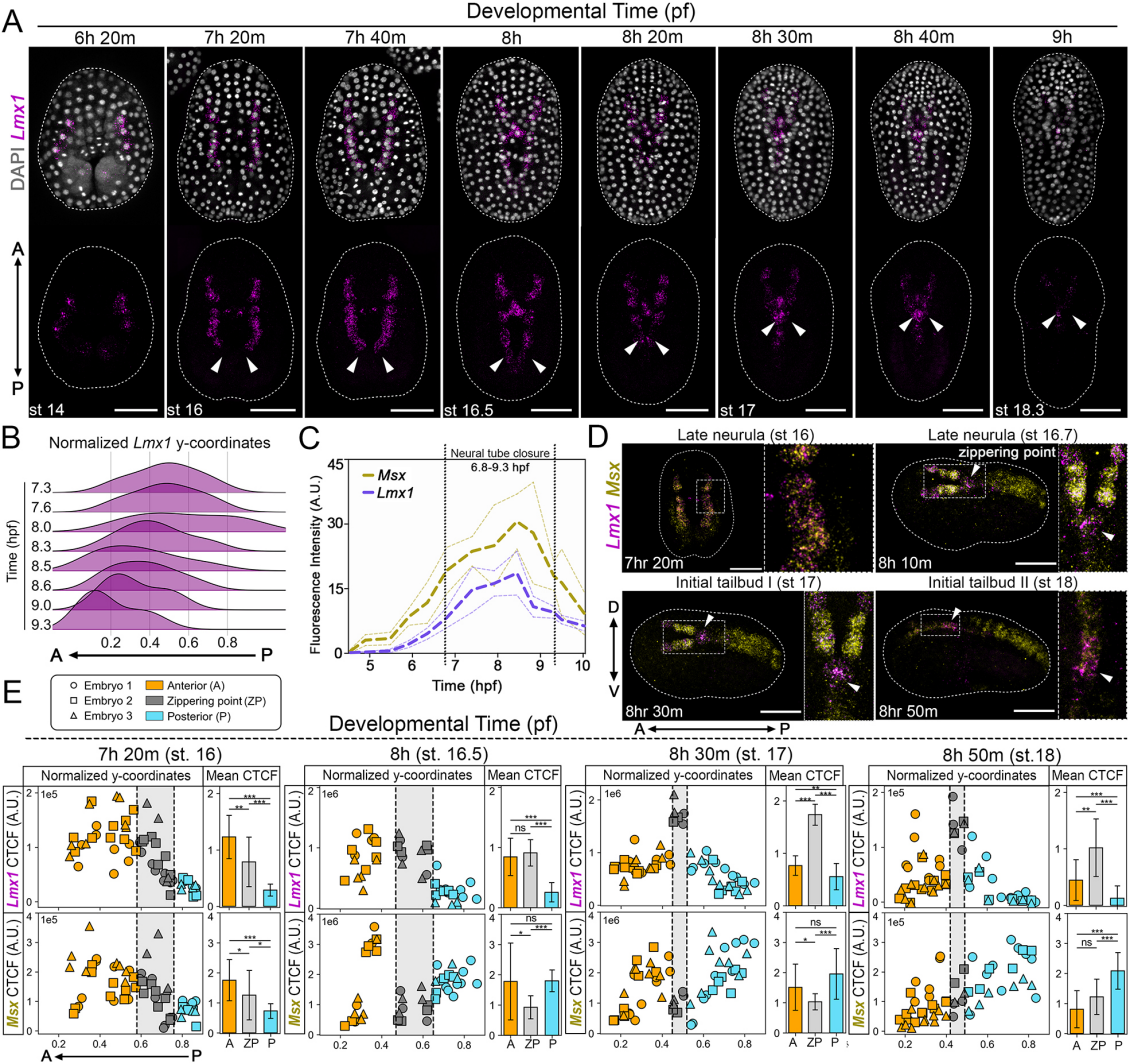
***Lmx1* exhibits a posterior-anterior transcription wave during neural tube zippering.** (A) Representative embryos showing HCR *in situ* hybridization signal for the *Lmx1* probe (magenta) and DAPI-stained nuclei (gray) during neurulation. *Lmx1* is expressed in the cells at the head of the zipper and in cells anterior to the zipper. Arrowheads indicate the zippering point. Dashed lines mark embryo outlines. Numbers of embryos examined: *n*=15 per experiment (three experiments). (B) Ridgeline plot of *y*-coordinates of *Lmx1* expression normalized to the embryo bounds for time points shown in A, demonstrating the posterior-to-anterior progression of *Lmx1* expression throughout neurulation (7.3-9.3 hpf; *n*=3 embryos per time point). (C) Quantification of total *Lmx1* and *Msx* transcriptional activity throughout gastrulation and neurulation (4.5-10 hpf). Dashed lines (bold) represent mean fluorescence intensity (*n*=5 embryos per time point); dashed lines (thin) indicate the standard deviation. (D) HCR images showing *Lmx1* (magenta) and *Msx* (yellow) expression at the dorsal midline reveal overlapping domains at 7 hpf and inverse expression at 8-9 hpf. Arrowheads indicate the zippering point. Dashed lines mark embryo outlines. Insets show magnification of boxed areas. Numbers of embryos examined: *n*=20 per experiment (three experiments). (E) Corrected total cell fluorescence (CTCF) measurements of *Lmx1* and *Msx* HCR signals in dorsal midline cells during NTC, plotted relative to the *y*-coordinate of each cell normalized to the embryo bounds (*n*=3 embryos per time point). Each point represents a single cell. Data show local enrichment of *Lmx1* at the zippering point (gray) and corresponding downregulation of *Msx* in the same region. Bar graphs show mean CTCF for cells anterior (orange), at (gray) and posterior (blue) to the zipper. Error bars indicate the standard deviation. ***P<0.001, **P<0.01, *P<0.05 (unpaired, two-tailed Welch's *t*-test, independent samples, with Holm-Bonferroni correction for multiple comparasions). n.s., not significant (≥0.05). Developmental stages and hpf are indicated in the images. Brightness and contrast of images were adjusted linearly. Scale bars: 50 μm.

Previous studies have identified *Lmx1* expression along the future dorsal midline during neurulation, but with limited spatial and temporal resolution (Ishida and Satou, 2024; Imai et al., 2006, 2004). Using high-resolution hybridization chain reaction (HCR) *in situ* hybridizations of precisely staged *Ciona* embryos, we found that *Lmx1* transcripts are not uniformly distributed but instead exhibit a posterior-to-anterior transcription wave during NTC (Fig. 1A,B). *Lmx1* is first detectable at the early gastrula stage in the future neural plate border (Fig. S1), peaks toward the end of neurulation (Fig. 1C) and subsequently declines. A ridgeline plot of the normalized *x*- and *y*-coordinates of *Lmx1*-expressing nuclei along the anteroposterior axis reveals a forward-shifting wave of nuclear transcripts (Fig. 1B). Notably, *Lmx1* expression is the highest at the zippering point and in anterior neural cells poised to fuse but reduced in posterior cells that have already undergone closure (Fig. 1D). This transcription wave coincides with the progression of zippering, suggesting that *Lmx1* activation either precedes or accompanies neural fold fusion and is tightly coupled to the morphogenetic events of zippering.

We next examined the expression of *Msx* (Movie 1), a conserved dorsal neural plate border marker in both *Ciona* and vertebrates (Ramos and Robert, 2005; Imai et al., 2004). During early neurulation, *Msx* and *Lmx1* are broadly co-expressed along the dorsal midline (Fig. 1D; Figs S1A-G and S2A). However, as the neural folds elevate and zipper, their expression becomes spatially refined (Fig. 1D). At the zippering point, *Lmx1* becomes enriched (Fig. S2B-D) while *Msx* is selectively downregulated (Fig. S2B,C), resulting in a locally exclusive pattern (Fig. 1E; Figs S1H-L and S2B,C). This transition indicates a spatially coordinated interplay between these transcriptional programs. The temporal handoff between *Lmx1* and *Msx* might reflect a regulatory switch, transitioning from *Msx*-guided dorsal patterning to *Lmx1*-mediated morphogenesis.

### *Lmx1* promotes cell proliferation at the dorsal midline

**Fig. 2.**
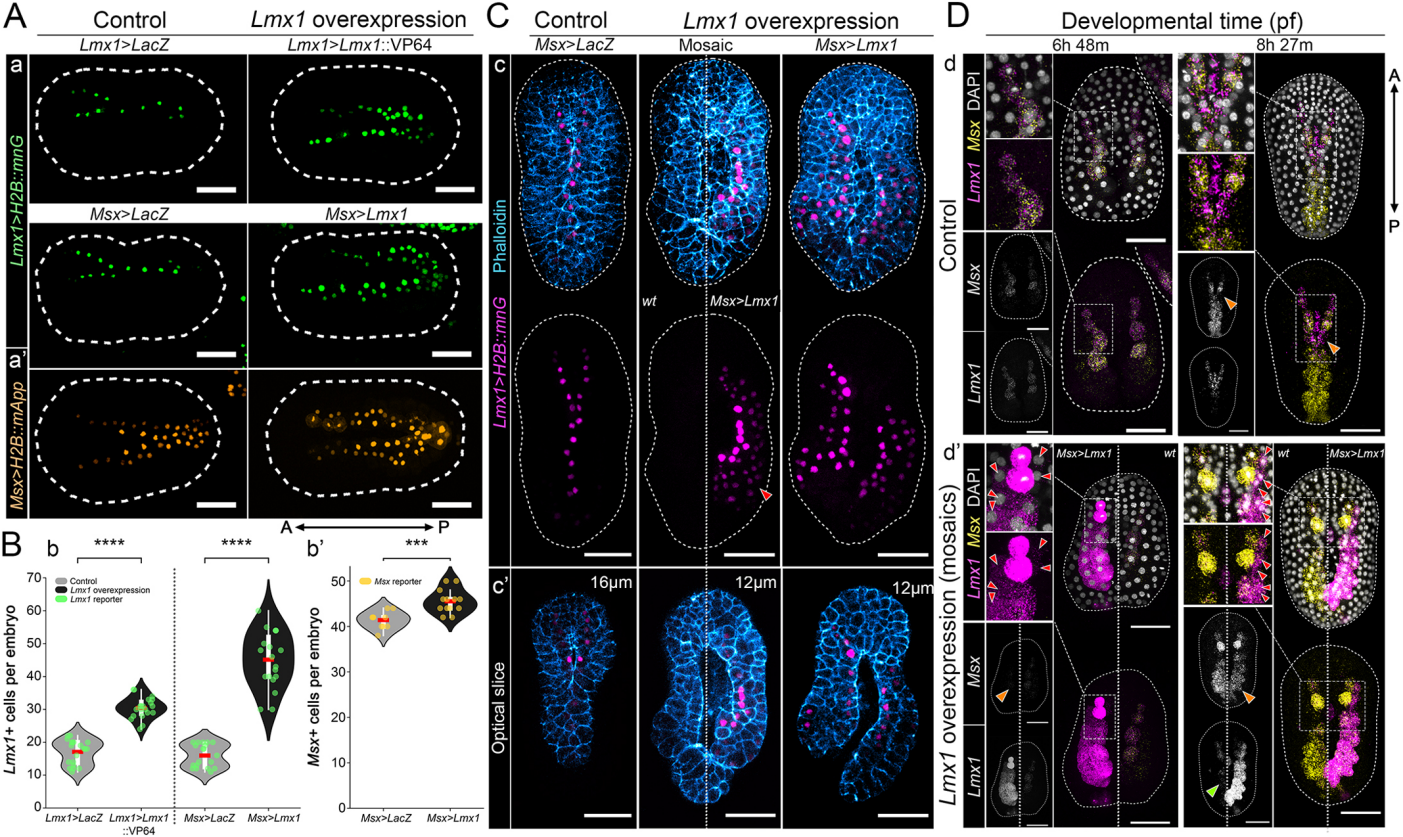
***Lmx1* autoregulates and promotes cell proliferation during neural tube closure.** (A) Representative images of initial tailbud I embryos expressing (Aa) an *Lmx1* nuclear reporter (*Lmx1*>*H2B::mnG*, green) and a control construct (*Lmx1*>*LacZ*, top left) or overexpressing *Lmx1* via fusion to a VP64 activation domain (*Lmx1*>*Lmx1::VP64*, top right), or with constructs under the *Msx* regulatory sequence (*Msx*>*LacZ*, bottom left; *Msx*>*Lmx1*, bottom right). (Aa′) *Msx* nuclear reporter (*Msx*>*H2B::mApp*, orange), with either control (*Msx*>*LacZ*, left) or overexpression (*Msx*>*Lmx1*, right). (B) Violin plots with overlaid box plots showing the number of dorsal midline cells expressing the nuclear reporters (Bb) *Lmx1*>*H2B::mnG* or (Bb′) *Msx*>*H2B::mApp* under the corresponding conditions shown in A. Each point represents the total number of reporter-positive nuclei per embryo; *n*=3220 total nuclei across 108 embryos. ****P≤0.0001, ***P≤0.001 (unpaired, two-tailed Welch's *t*-test, independent samples). Box plots inside violin plots show the interquartile range (IQR; white boxes) with whiskers extending to 1.5× the IQR; red bars indicate the mean. (C) Representative maximum intensity projections of initial tailbud I images of embryos expressing *Lmx1*>*H2B::mnG* (magenta), and either *Msx*>*LacZ* or *Msx*>*Lmx1*, with F-actin (blue). Expression of *Msx*>*Lmx1* and *Lmx1*>*H2B::mnG* is mosaic and restricted to the right side of the embryo (middle). (Cc′) Optical slices from *z*-stacks in (Cc), taken at 16 μm (left) and 12 μm (middle, right) from the dorsal surface, illustrate both the cell-autonomous effects of *Lmx1* overexpression and its impact on NTC. Numbers of embryos examined: *n*=20 per experiment (three experiments). (D) Representative HCR images at the mid neurula (6 h 48 min pf) and initial tailbud I (8 h 27 min pf) stages showing *Lmx1* (magenta) and *Msx* (yellow) probe; DAPI-stained nuclei (gray). (Dd) control embryos expressing *Msx*>*LacZ*; (Dd′) mosaic overexpression of *Lmx1* (*Msx*>*Lmx1*) on the left (6 h 48 min pf) and right (8 h 27 min pf) sides of the embryo. Orange arrowheads indicate *Msx* repression coinciding with *Lmx1* enrichment. Red arrowheads indicate ectopic activation of *Lmx1* in neighboring cells outside of the *Msx* domain, suggesting non-cell-autonomous activity. Green arrowhead indicates loss of the *Lmx1* transcription wave and zippering failure. Numbers of embryos examined: *n*=20 per experiment (three experiments). Brightness and contrast of images were adjusted linearly. White dashed lines represent the embryo outline. Gray dashed lines represent insets of embryos. Scale bars: 50 μm.

To test the function of *Lmx1* during NTC, we overexpressed *Lmx1* under the control of the *Msx* regulatory sequence. This led to an expanded dorsal midline domain (Fig. 2A), significantly increased numbers of *Lmx1*- and *Msx*-reporter-positive nuclei (Fig. 2B), and impaired zippering (Fig. 2C,D). Phalloidin staining revealed disruptions to apical seam organization (Fig. 2C), suggesting that elevated cell density interferes with neural fold fusion, possibly by perturbing the spatial constraints necessary for coordinated cell movements.

To determine whether these effects reflect an *Lmx1*-specific role rather than ectopic activation by the *Msx* enhancer, we also overexpressed a transcriptionally activated form, *Lmx1::VP64*, under the control of the *Lmx1* enhancer (Fig. 2A). This construct similarly increased the number of *Lmx1*-reporter-positive nuclei (Fig. 2B), suggesting that *Lmx1* promotes proliferation within the *Lmx1*-expression domain. However, we cannot conclusively determine whether all additional cells originate from overproliferation (see below).

In embryos overexpressing *Lmx1* under the control of the *Msx* enhancer, an *Lmx1* reporter gene was ectopically activated in the dorsal epidermis, outside of its normal domain of expression (Fig. 2C). This demonstrates that *Lmx1* is sufficient to activate its own enhancer in a cell-autonomous manner, consistent with an autoregulatory feedback loop. This autoregulatory capacity may reinforce *Lmx1* expression in zippering cells and sustain the proliferative state. Importantly, in mosaic embryos, we observed that *Msx>Lmx1* overexpression led to activation of *Lmx1* in neighboring cells that did not express *Msx* (Fig. 2D′; red arrowheads), suggesting non-autonomous expansion of *Lmx1* expression. These findings support an *Lmx1* transcriptional wave model, whereby *Lmx1* not only reinforces its own expression through direct positive feedback but also amplifies its spatial domain through local cell-cell interactions. This behavior suggests that *Lmx1* sits at the core of a dynamic transcriptional circuit that integrates proliferation, patterning and mechanical coordination during NTC.

In these same mosaic embryos, *Lmx1* overexpression led to local downregulation of *Msx* in *Lmx1*-overexpressing cells (Fig. 2D; orange arrowheads), consistent with repressive feedback from downstream components described in previous studies (Roure and Darras, 2016), and may suggest that *Lmx1* participates directly or indirectly to repress *Msx* expression and thereby promote morphogenetic progression. Notably, the endogenous *Lmx1* transcriptional wave was disrupted (Fig. 2Dd′; green arrowhead), suggesting that either the wave is necessary for zippering or that ongoing zippering is required to maintain *Lmx1* activation dynamics. Taken together, these findings show that *Lmx1* reinforces its own expression cell-autonomously, and might also contribute to the expansion of the proliferative domain. This behavior is consistent with a positive feedback loop operating within a bistable or excitable system (Zhao et al., 2023) and underscores the importance of precisely patterned *Lmx1* expression for robust morphogenesis.

### *Cdkn1b* restricts proliferation and contributes to proper zippering dynamics

Given that *Lmx1* might promote proliferation, we next asked whether *Cdkn1b*, a known cell-cycle inhibitor (Treen et al., 2023b Kobayashi et al., 2022), acts as a counterbalance during NTC. High-resolution HCR *in situ* hybridization enabled us to visualize the spatiotemporal dynamics of *Cdkn1b* expression throughout neurulation (Figs S3 and S4). We found that *Cdkn1b* is expressed globally throughout neurulation, with enrichment along the dorsal midline during anterior closure (Fig. S4E-K). Notably, analysis of individual confocal *z*-planes through the roof plate revealed a striking alternating pattern of *Cdkn1b* expression in neural cells at the dorsal midline, with adjacent cells showing either high or undetectable levels. This pattern mirrored that of *Lmx1* and suggested a spatially coordinated transcriptional program.

**Fig. 3.**
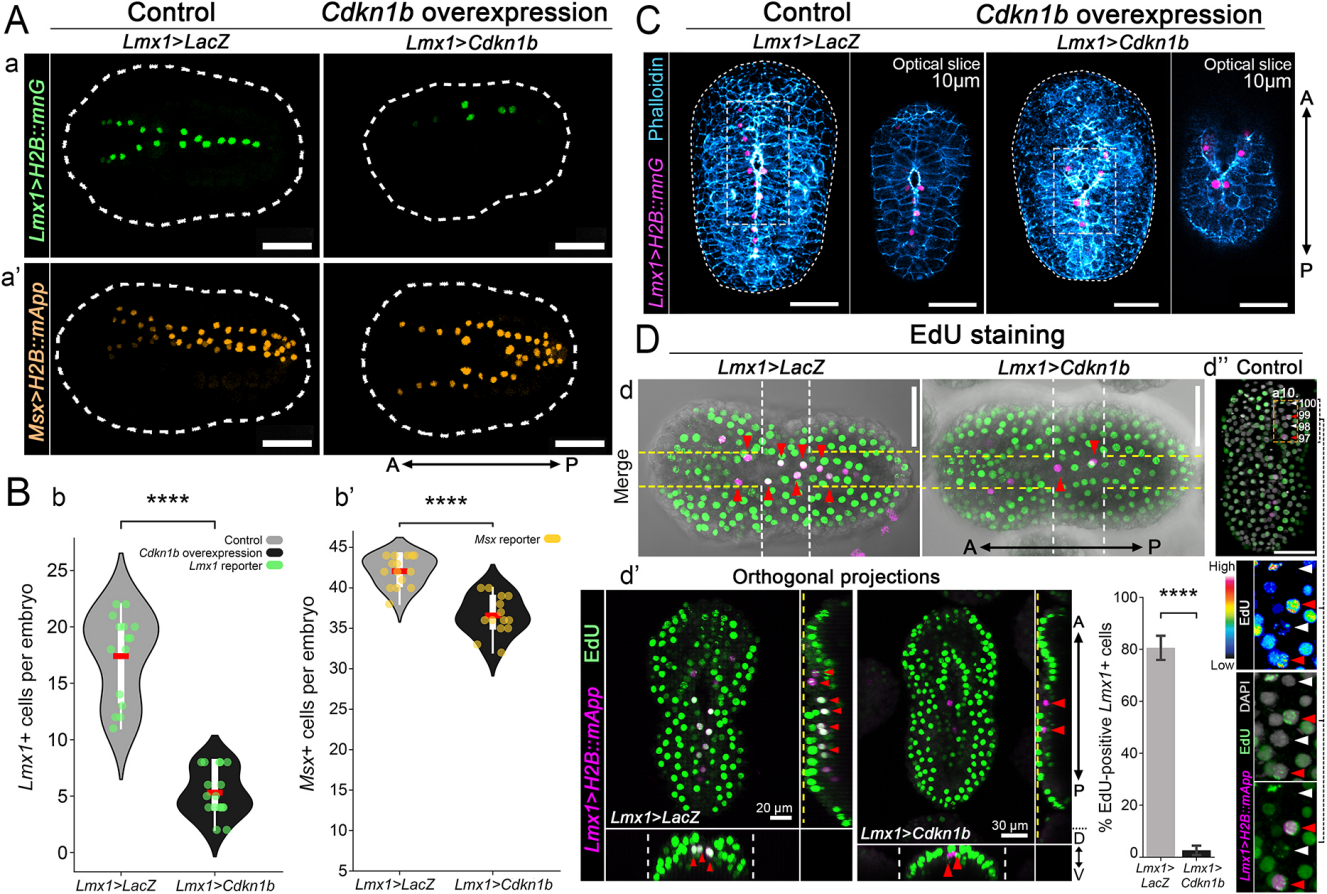
***Cdkn1b* involvement in cell cycle regulation during neural tube closure.** (A) Representative images of initial tailbud I stage embryos expressing (Aa) an *Lmx1* nuclear reporter (*Lmx1*>*H2B::mnG*, green) with either a control construct (*Lmx1*>*LacZ*) or overexpressing *Cdkn1b* under the *Lmx1* regulatory sequence (*Lmx1*>*Cdkn1b*); (Aa′) *Msx* nuclear reporter (*Msx*>*H2B::mApp*, orange) with either *Lmx1*>*LacZ* or *Lmx1*>*Cdkn1b*. (B) Violin plots with box plots of the number of dorsal midline cells expressing (Bb) *Lmx1*>*H2B::mnG* or (Bb′) *Msx*>*H2B::mApp* under the corresponding conditions shown in A. Each point represents the total number of reporter-positive nuclei per embryo; *n*=1526 total nuclei examined across 15 embryos per condition (unpaired, two-tailed Welch's *t*-test, independent samples: ****P≤0.0001, ***P<0.001). Box plots inside violin plots show the IQR (white boxes) with whiskers extending to 1.5× the IQR; red bars indicate the mean. (C) Representative maximum intensity projections of *z*-stacks showing embryos expressing *Lmx1*>*H2B::mnG* (magenta), *Lmx1*>*LacZ* (left) or *Lmx1*>*Cdkn1b* (right), with F-actin (blue). White dashed lines outline embryo boundaries. Gray dashed lines outline insets corresponding to the optical sections (10 μm from the dorsal surface) highlighting the effects of *Cdkn1b* overexpression on NTC. Numbers of embryos examined: *n*=20 per experiment (three experiments). (D) EdU labeling (green) of embryos expressing the *Lmx1* nuclear reporter *Lmx1*>*H2B::mApp* (magenta), and either *Lmx1*>*LacZ* or *Lmx1*>*Cdkn1b*. (Dd) Maximum intensity projections merged with differential interference contrast (DIC). (Dd′) orthogonal projections showing the *xz*-plane (white dashed line; bottom), and *yz*-plane (yellow dashed line; right) of embryos in Dd, showing active proliferation in controls but almost complete absence in *Cdkn1b*-overexpressing embryos, confirming that *Cdkn1b* arrests the cell-cycle in dorsal midline cells. Bar graph shows mean percentage EdU^+^ cells in the *Lmx1* domain shown in D. ****P<0.0001 (Mann–Whitney *U*-test, two-tailed). Error bars indicate s.e.m.; *n*=337 total nuclei examined across 15 embryos per condition. (Dd″) EdU labeled embryos counterstained with DAPI (gray) showing high EdU levels in *Lmx1*^+^ cells (a10.97 and a10.99; red arrowheads) and low levels in adjacent *Lmx1*^−^ cells (a10.98 and a10.100; white arrowheads). Dashed line (orange) indicates the insets. Brightness and contrast were adjusted linearly. Scale bars: 50 μm (unless otherwise noted).

To test *Cdkn1b* function, we overexpressed it under the control of the *Lmx1* regulatory sequence, restricting expression to dorsal midline neural cells. Compared to *LacZ* controls, *Cdkn1b*-overexpressing embryos were shorter (Fig. 3A), had a reduced number of neural tube cells (Fig. 3B) and exhibited stalled NTC (Fig. 3C).

**Fig. 4.**
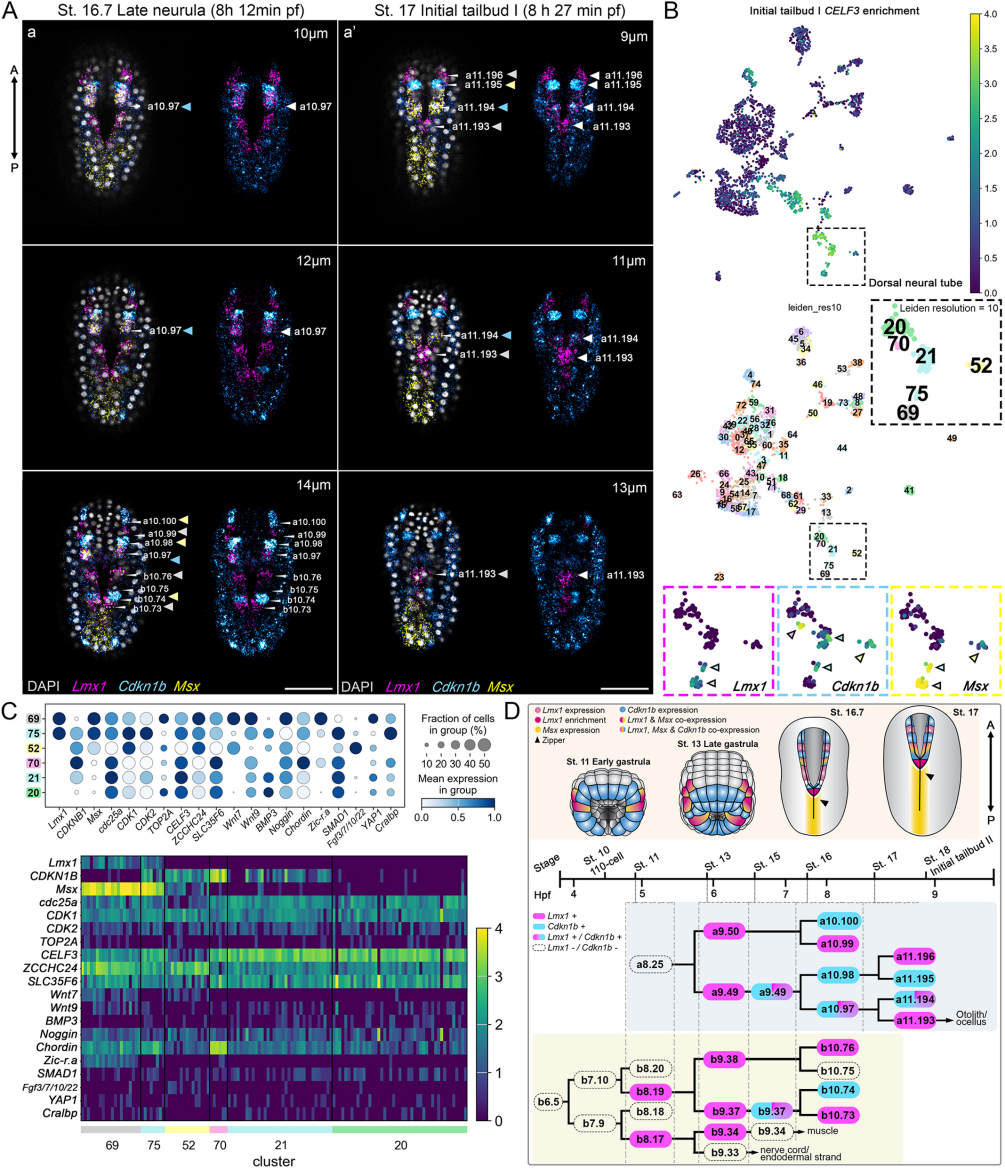
***Lmx1* and *Cdkn1b* exhibit a pair-rule-like alternating expression during anterior neural fold fusion, suggesting patterned transcriptional control of neural fold fusion during closure.** (A) Representative optical slices from *z*-projected stacks of (Aa) late neurula (8 h 12 min pf) embryos at depth 10-14 μm, and (Aa′) initial tailbud I (8 h 27 min pf) embryos at depth 9-13 μm from the dorsal surface, reveal the pair-rule-like expression pattern outlined in D, where *Lmx1* and *Cdkn1b* show an alternating expression pattern in dorsal midline cells that are zippering during anterior NTC. HCR *in situ* hybridization signals for *Lmx1* (magenta), *Msx* (yellow) and *Cdkn1b* (cyan) probe; DAPI-stained nuclei (gray). (B) Representative UMAP projection of single-cell transcriptomes at the initial tailbud I stage color-coded with the expression level of the neural marker *CELF3* (Yagi and Makabe, 2001; Satou et al., 2001) and annotated by Leiden clusters (resolution=10). Cluster identities are indicated with pastel hues. Roof plate clusters (20, 21, 52, 69, 70) are highlighted in the subpanel. Expression of *Lmx1*, *Cdkn1b* and *Msx* across clusters is shown in adjacent panels, revealing transcriptionally defined populations consistent with the pair-rule-like pattern observed *in vivo*. (C) Dot plot shows relative expression levels of genes across clusters in B, chosen for relevance to neural identity, cell-cycle regulation and epithelial remodeling. The heatmap illustrates their expression across individual cells. (D) Schematic representation of *Lmx1* and *Cdkn1b* expression patterns from gastrulation to neurulation. Arrowheads indicate: (1) dorsal midline cells (white) highlighted in magenta or cyan in panel (A) and in the lineage map (D) corresponding to b8.17 and b8.19 neural plate border cells and the a9.49-derived cells, the rudimentary neural crest lineage; and (2) the neural clusters in the UMAP (B) to which these cells likely correspond (cluster 69, gray; 75, blue; 52, yellow). Brightness and contrast were adjusted linearly. Number of embryos examined: *n*=15 per experiment (five experiments). Scale bars: 50 μm.

To directly assess the effect of *Cdkn1b* on cell-cycle progression, we performed EdU incorporation assays in embryos co-expressing an *Lmx1* nuclear reporter with either *Lmx1*>*LacZ* or *Lmx1*>*Cdkn1b*. In controls, EdU^+^ nuclei were readily detected within the *Lmx1*-domain, whereas EdU labeling was nearly absent in *Cdkn1b*-overexpressing embryos, confirming that *Cdkn1b* arrests S-phase entry in dorsal midline cells (Fig. 3D). Furthermore, EdU-labeled embryos counterstained with DAPI revealed that strongly EdU^+^ nuclei correspond to *Lmx1*^+^ cells (a10.97 and a10.99), while *Lmx1*^−^ neighboring cells (a10.98 and a10.100) showed little or no EdU incorporation (Fig. 3Dd″), consistent with alternating cell-cycle states among anterior neighboring cells in the dorsal midline. This lineage-level alternation parallels the complementary pattern of *Lmx1* and *Cdkn1b* transcripts revealed by multiplex HCR, with *Lmx1* enriched in a10.99, *Cdkn1b* in a10.98 and a10.100, and co-expression of *Lmx1* and *Cdkn1b* restricted to the a10.97-derived rudimentary neural crest lineage (Fig. 4A).

Unlike *Lmx1* overexpression, which disrupted apical F-actin, *Cdkn1b*-overexpressing embryos retained apical seam integrity at the zippering point but exhibited reduced dorsal tissue thickness due to diminished proliferation (Fig. 3C). These observations imply that impaired closure in *Cdkn1b*-overexpressing embryos reflects insufficient cell number rather than defects in cytoskeletal organization. These findings reveal that neural fold fusion is constrained by a biophysical threshold of local cell density below or above which fusion fails to proceed efficiently and that *Cdkn1b* may help modulate this threshold. Together, these results point to a finely tuned, transcriptionally regulated coordination of cell-cycle dynamics in both neural and epidermal tissues during neurulation.

### *Lmx1* and *Cdkn1b* exhibit a pair-rule-like expression pattern during anterior NTC

To investigate the spatial relationship between *Lmx1* and *Cdkn1b* expression, we performed multiplex HCR *in situ* hybridizations. We found that these genes exhibited strikingly complementary expression patterns across the dorsal midline, with *Lmx1* and *Cdkn1b* localized to adjacent, non-overlapping cells (Fig. 4A). This alternating, pair-rule-like motif was especially prominent in the anterior neural folds during active zippering (Fig. 4A), suggesting that *Lmx1* and *Cdkn1b* define transcriptionally distinct anti-proliferative (*Cdkn1b*) and permissive (*Lmx1*) regulatory regimes within a shared morphogenetic field. The emergence of this pattern coincides temporally with peak zippering behavior, raising the possibility that alternating transcriptional states serve to modulate cell density and behavior at the cellular scale, reminiscent of pair-rule patterning logic in other systems (Clark et al., 2019; Nüsslein-Volhard and Wieschaus, 1980).

To test this idea, we analyzed previously published single-cell RNA-seq data (Cao et al., 2019) from initial tailbud I embryos (Fig. 4B). Within the neural lineage, *Lmx1* and *Cdkn1b* expression mirrored the mutually exclusive patterns observed *in vivo*. High-resolution clustering (Leiden resolution=10) further revealed three transcriptionally distinct dorsal neural populations (Fig. 4B). These include: (1) *Lmx1^+^*/*Msx*^+^ cells enriched for proliferative markers such as *cdc25a* and *CDK1* (Fig. 4C; cluster 69); (2) *Msx^+^*/*Cdkn1b^+^* cells enriched for differentiation and signaling markers such as *Fgf3/7/10/22* (Fig. 4C; cluster 52): and (3) a mixed population co-expressing all three (Fig. 4C; cluster 75) in addition to the ependymal marker *Cralbp* (also known as *Rlbp1*), which may indicate a transitional progenitor pool, potentially contributing to the rudimentary neural crest. This suggests a regulatory switch where cell-cycle exit and differentiation are tightly coupled to lineage identity.

*In vivo*, co-expression of *Lmx1*, *Msx* and *Cdkn1b* was restricted to the a10.97 lineage (Fig. 4A,D), previously shown to give rise to the rudimentary neural crest lineage in *Ciona* (Todorov et al., 2024). Outside this lineage, *Lmx1* and *Cdkn1b* expression remained largely mutually exclusive, reinforcing the idea that their alternation reflects a functional division between proliferative and differentiating states. This complementary patterning may help buffer against mechanical or proliferative noise by distributing cell states in a spatially interdigitated fashion, thereby enhancing the robustness of zippering. Together, these findings suggest that a pair-rule-like transcriptional logic contributes to dorsal midline organization during NTC.

## DISCUSSION

This study identified a previously unreported transcriptional framework for coordinating cell-cycle state and morphogenetic behavior during NTC in *Ciona*. We show that *Lmx1* might function as a proliferative transcription factor expressed in a dynamic posterior-to-anterior transcription wave at the zippering point. *Lmx1* is sufficient to activate its own expression, expands the local proliferative domain and is involved in neural fold fusion. In contrast, the cell-cycle inhibitor *Cdkn1b* acts as a spatially restricted brake on proliferation, expressed in an alternating pattern with *Lmx1*. This complementary expression balances cell density across the neural folds during anterior NTC.

A key feature of this system is a temporal handoff between *Msx* and *Lmx1* during NTC. Early in neurulation, *Msx* and *Lmx1* are co-expressed in the neural folds but, as closure progresses, *Msx* becomes selectively downregulated at the zippering point while *Lmx1* is upregulated. Our data indicate that this transition reflects active *Msx* repression. Our findings show that *Lmx1* overexpression leads to local downregulation of *Msx* (Fig. 2D; orange arrowheads), which is consistent with cell-autonomous negative feedback. This observation is aligned with previous gene regulatory network reconstructions in *Ciona* that identified widespread negative feedback onto *Msx* from its downstream targets (Roure and Darras, 2016). The expression of *Lmx1* in *Msx*^−^ cells marks a transition from early patterning to morphogenetic activity, where cells begin to proliferate and intercalate. The mutually exclusive expression indicates a transcriptional switch that gates entry into a proliferative, mechanically permissive state, positioning *Msx*-to-*Lmx1* transition as a key regulatory node in coordinating spatial organization during NTC.

The interleaved expression of *Lmx1* and *Cdkn1b* evokes the pair-rule patterning logic classically associated with arthropod segmentation (Zallen and Wieschaus, 2004; Irvine and Wieschaus, 1994). By alternating cycling and arrested cells, this transcriptionally patterned landscape may prevent mechanical jamming and allow more efficient fusion. The transient block on the cell-cycle due to the expression of *Cdkn1b* might be necessary to prevent cell division in front of the zippering point, which would lead to its arrest as observed with the adjacent epidermis (Ogura and Sasakura, 2016; Ogura et al., 2011). Computational models in other systems have previously shown that mechanical heterogeneity, typically achieved via differential tension and cell-cycle state, enhances robustness in tissue closure (Bocanegra-Moreno et al., 2023; Kim et al., 2021; Angelini et al., 2011). Our findings provide empirical evidence for such a mechanism in a chordate. Additionally, the ability of vertebrate LMX1 paralogs to promote proliferation in the midbrain indicates their potential involvement in its regulation within the neural folds and the roof plate (Yan et al., 2011). It is possible that pair-rule segmentation in *Drosophila* and other arthropods establishes similar differential proliferative states to foster morphogenetic processes such as germband elongation (Clark et al., 2019).

Our perturbation data highlight the importance of spatially tuned proliferation. Overexpression of *Lmx1* leads to dorsal crowding, disrupted F-actin polarity and failed zippering. This is consistent with mechanical jamming, a condition where excess cell density in conjunction with tension fluctuations inhibits coordinated movement due to overcrowding, reducing tissue fluidity (Sakamoto et al., 2023; Kim et al., 2021). Conversely, *Cdkn1b* overexpression reduces neural cell number and stalls NTC, despite preserved cytoskeletal organization. These embryos may experience mechanical under-loading, where insufficient traction or cell-cell contact prevents the generation of forces needed to drive zippering. This aligns with known previous observations of decreases in proliferation rate leading to a decline in cell rearrangements and tissue growth rate (Bocanegra-Moreno et al., 2023). These outcomes suggest that neural fold fusion efficiency operates within a ‘Goldilocks’ zone of cell density, modulated by opposing cues.

Mechanistically, the *Lmx1-Cdkn1b* circuit might regulate both proliferation and biomechanical properties of zippering cells. The capacity of *Lmx1* to activate its own expression both cell-autonomously and potentially non-autonomously suggests a positive feedback loop stabilizing proliferative domains across the neural folds. Moreover, the mutual exclusivity of *Lmx1* and *Msx* at the zippering point further supports the idea of a bistable transcriptional switch coordinating morphogenetic transitions.

Single-cell RNA-seq analysis corroborates this framework, revealing three distinct dorsal neural transcriptional states linked to specialized roles during NTC. One cluster co-expresses *Lmx1* and *Msx* (Fig. 4C; cluster 69) and also exhibits high expression levels of *Wnt7*/*9*, *Noggin*, *YAP1* and cell-cycle drivers (*cdc25a*, *CDK1* and *TOP2A*) but essentially lack *Cdkn1b*. This transcriptional profile is characteristic of proliferative and mechanosensitive fusing of the neural folds, forming one-half of the pair-rule-like pattern where alternating cells drive closure. A second cell cluster uniquely co-expresses *Lmx1*, *Cdkn1b* and *Msx* (Fig. 4C; cluster 75) along with *Cralbp* and moderate levels of *Wnt9*, *Chordin* and *SMAD1*. This may represent a transitional progenitor pool with bistability properties as these cells express both proliferative and inhibitory cell-cycle markers. The third cell cluster co-expresses *Cdkn1b* and *Msx* (Fig. 4C; cluster 52) and is characterized by high *Fgf3/7/10/22* (the closest human gene is *FGF3*) expression and an absence of *Lmx1*, *Zic-r.a* and *YAP1*, marking a transition from dorsal progenitor identity to a post-mitotic, differentiating neural fate. Although the expression of *CDK1* remains high, the reduced expression of *CDK2* and *TOP2A* suggests that cells are withdrawing from the cell-cycle or entering arrest. This population likely represents the complementary half of the pair-rule architecture, contributing structural stability during NTC. Together, these data suggest a transcriptional landscape with intricate links between cell-cycle dynamics, patterning state and differentiation.

### Conclusion

We report a striking pair-rule-like transcriptional logic associated with proliferation and morphogenesis during NTC. The alternating expression of *Lmx1* and *Cdkn1b* delineates distinct proliferative regimes, while a temporal handoff from *Msx* to *Lmx1* marks the transition from early patterning to morphogenetic execution. This transcriptional architecture likely ensures robust neural fold fusion by spatially organizing mechanical forces and proliferation and may represent a conserved strategy across metazoans for integrating fate, behavior and tissue mechanics.

## MATERIALS AND METHODS

### *Ciona* handling, embryo collection and electroporation

Adult *Ciona intestinalis* (type A; also called *Ciona robusta*) were obtained from Marine Research and Educational Products and Marinus Scientific and maintained in artificial seawater at 18°C. Eggs and sperm were harvested and dechorionated using a solution containing 60 mM NaOH, 1% (w/v) sodium thioglycolate and 0.1% (w/v) Actinase E in artificial seawater for 10 min, as previously described (Christiaen et al., 2009b). Dechorionated eggs were then washed four times with artificial seawater before self-fertilization by incubation with sperm for 15 min, followed by two additional washes. All embryos used in this study are biological replicates.

One-cell stage embryos were transfected by electroporation with plasmid DNA, in accordance with a previously described method (Christiaen et al., 2009a). Transgenic embryos were cultured in artificial seawater on gelatin-coated dishes at 18°C until the desired developmental stage (Hotta et al., 2007). Embryos destined for cytoskeletal visualization were then fixed in MEM-formaldehyde (4% formaldehyde, 100 mM MOPS pH 7.4, 500 mM NaCl, 1 mM EGTA, 2 mM MgSO_4_ and 0.05% Tween-20 in ultrapure H_2_O) for 30 min at room temperature, followed by three washes in PBST (phosphate buffer solution with 0.1% Tween-20). To visualize the cytoskeleton, embryos were incubated with Alexa Fluor 555-phalloidin (Abcam; 40× stock, used at 1:50 dilution) for 30 min and then washed three times with PBST.

### Molecular cloning

The reporters *Lmx1>H2B::mnG* and *Lmx1>H2B::mApp* were previously described (Todorov et al., 2024). The *Msx* and *Sox1/2/3* regulatory sequences were published by Abitua and colleagues (Abitua et al., 2012) and Khoueiry and colleagues (Khoueiry et al., 2010), respectively. They were subcloned into a *H2B::mApp* expression plasmid (Cao et al., 2019) using *NotI* and *AscI* restriction enzymes (New England Biolabs). Control constructs (*Lmx1>LacZ* and *Msx>LacZ*) were obtained by subcloning *Lmx1* (Lemaire et al., 2021) and *Msx* regulatory sequences into *LacZ* expression plasmids (Fujiwara et al., 1998) using *NotI* and *AscI* restriction enzymes (New England Biolabs).

The plekstrin homology domain of human *PLCD1* fused to *neongreen* (*PH-nG*) expression vector described in Cao et al. (2019) was used to generate the *Msx 1.5 kb*>*PH::mnG*. An additional degradation signal was added to destabilize the membrane fluorescent reporter protein further. For this, the degradation signal from the *PH-nG* expression vector was PCR-amplified and inserted by recombination (NEBuilder, New England Biolabs) into the same vector digested with the *SpeI* restriction enzyme (New England Biolabs). The regulatory sequence *Msx* 1.5 kb (KY21.Chr2:6131940..6133585) was PCR-amplified from an *Msx* reporter and cloned into the obtained expression vector using the *NotI* and *AscI* restriction enzymes (New England Biolabs), followed by ligation (T4 DNA ligase, Promega).

The regulatory sequences of *Lmx1* (2029 bp; KY21.Chr9:4348500..4350529) and *Msx* (2446 bp; KY21.Chr2:6131140..6133586) were PCR-amplified from the plasmids *Lmx1>H2B::mnG* and *Msx>H2B::mApp*. Reporter and overexpression constructs were generated via Gibson assembly using PCR-amplified fragments with designed overhangs (primer sequences provided in Table S3). To generate *Lmx1>GFP*, the *Lmx1* regulatory sequence was PCR-amplified from *Lmx1>H2B::mnG* and the *GFP* coding sequence (717 bp) from the *TRE3G-GFP* vector (gift of Dr Charles Ettensohn, Carnegie Mellon University, Pittsburgh, PA, USA). For *Lmx1>Cdkn1b*, the *Lmx1* regulatory sequence was paired with the *Cdkn1b* coding sequence (915 bp) amplified from *Mesp>CkiB* (gift of Dr Nicholas Treen, Princeton University, NJ, USA). To create *Msx>Lmx1*, the *Msx* regulatory sequence was combined with the *Lmx1* coding sequence amplified from cDNA obtained by retrotranscription using Superscript II Reverse Transcriptase (Invitrogen) of 800 ng *Ciona* neurula RNA extracted using Qiagen RNA Extraction Kit. Transgenic constructs were assembled into circular plasmids using HiFi DNA Assembly (New England Biolabs).

The transgene expressing the constitutively active *Lmx1* transcription factor controlled by *Lmx1* regulatory sequences (*Lmx1*>*Lmx1::VP64*) was obtained by first cloning *Lmx1* regulatory sequences (Lemaire et al., 2021) into an expression vector containing the VP64 activation domain (Gainous et al., 2015) using the *NotI* and *AscI* restriction enzymes (New England Biolabs). The coding sequence of *Lmx1* was then PCR amplified, digested with *NotI* and *NaeI* restriction enzymes (New England Biolabs), and ligated (T4 DNA ligase, Promega) into the previously obtained plasmid digested with the same enzymes.

All constructs were validated by sequencing, and all functional assays were performed at least twice with batches of embryos harvested from different animals. Gene identifiers (KY21 gene model; Table S1) used in this study are as follows: KY21.Chr9.606 (*Lmx1*); KY21.Chr2.1031 (*Msx*); KY21.Chr2.18 (*Cdkn1b*); and KY21.Chr1.254 (*Sox1/2/3*).

### EdU staining

To detect proliferation, the modified thymidine analog EdU (5-ethynyl-2′-deoxyuridine) was incorporated into newly synthesized DNA using the Click-iT Plus EdU Alexa Fluor 488 imaging kit (Thermo Fisher Scientific). Embryos were treated with 10 µM EdU in seawater at 7.25 h post-fertilization (pf) for 1 h 10 min at 18°C. Following incubation, the EdU-labeled embryos were quickly fixed in 4% formaldehyde in PBST for 30 min, then washed twice in 3% bovine serum albumin (BSA) in PBS. Embryos were subsequently incubated in PBST at room temperature for 20 min, followed by two additional washes in 3% BSA in PBS. EdU detection was performed using a 30 min incubation in freshly made 1× Click-iT EdU reaction buffer at room temperature, protected from light. Finally, embryos were washed twice in 3% BSA in PBS, counterstained with DAPI (1 µg/ml, Thermo Fisher Scientific) and stored in PBST at 4°C before imaging.

### HCR *in situ* hybridization and imaging

Embryos were cultured in artificial seawater on gelatin-coated dishes at 18°C until the desired developmental stage (Hotta et al., 2007) and then fixed in EGS fixative (1% formaldehyde, 100 mM HEPES, 500 mM NaCl, 2 mM MgSO_4_, 2 mM EGS [ethylene glycol bis(succinimidyl succinate)] in ultrapure H_2_O) and stored at −20°C before staining. HCR *in situ* hybridization was performed using reagents and protocols provided by Molecular Instruments (sea urchin protocol), with minor modifications: the probe-hybridization buffer contained 50% formamide, 5× Denhart's, 0.01% (w/v) ssDNA, 0.01% (w/v) yeast DNA and 5× SSC (Treen et al., 2023a). The hairpin concentration was adjusted to 3 µM (2 µl hairpin in 100 µl amplification buffer) and hairpin amplification incubation time was shortened to 5 h. DAPI (1 µg/ml, Thermo Fisher Scientific) was added post-hybridization to visualize nuclei. Embryos were washed in 5× SSCT (5× SSC with 0.1% Tween-20) and stored in PBST at 4°C before imaging. Probe sets (probe sequences in Table S2) were designed by Molecular Instruments using the KY21 gene models: KY21.Chr9.606.v1.SL2-1 (*Lmx1*), KY21.Chr2.1031.v1.nonSL5-1 (*Msx*) and KY21.Chr2.18.v1.SL1-1 (*Cdkn1b*), compatible with amplifiers B1 (*Lmx1*), B3 (*Cdkn1b*) and B5 (*Msx*), respectively.

Samples were imaged using a Zeiss LSM 880 confocal microscope with a 20× objective, using Zen Black acquisition software. *Z*-stacks were acquired at 1 µm intervals, with a pixel size of 0.198×0.198 µm, and fluorophores were excited using 405, 488, 514, 561 and 633 nm lasers. Maximum intensity projections were generated in Zen Black and, unless otherwise stated, the images shown in this study represent these projections. The Imaris Viewer (10.2.0) was used to generate orthogonal projections.

Fluorescence intensity quantification was performed in FIJI/ImageJ v1.53k (Schindelin et al., 2012). Regions of interest (ROI) corresponding to nuclei or expression domains were manually defined using standardized shapes and sizes across embryos, ensuring consistency within and across experimental conditions and developmental stages. Signal intensities were quantified from raw, single-channel images (not pseudocolor overlays). All quantification was performed unaware of condition to avoid bias.

### Two-photon live imaging

For the time-lapse video acquisition with a two-photon microscope, one-cell-stage embryos were electroporated with 20 µg of *Msxb 1.5 kb*>*PH::mnG* and 40 µg of *Sox1/2/3*>*H2B::mApp*. Live imaging was performed as previously described (Todorov et al., 2024). Briefly, embryos were reared at 21°C. At the early neurula stage (stage 14), embryos were embedded into 1% methylcellulose in artificial seawater and placed individually in a 1% agarose microwell (Gregory and Veeman, 2013; Negishi et al., 2013). A custom-built upright two-photon microscope was used to image embryos until complete closure of their neural tube at 18°C. We processed the images with Fiji/ImageJ2 (v.2.14.0/1.54 f, ImageJ).

### Time-course quantification of HCR signal

To assess temporal changes in gene expression, HCR fluorescence was quantified from whole embryos across five embryos per time point (developmental stages 10-21) following a standardized thresholding workflow. Before measurement, each embryo was manually outlined using the polygon selection tool, and the background outside of the embryo was cleared to eliminate non-specific signals. Quantitation was then carried out using identical ROIs, positioned consistently across all embryos and timepoints. To isolate the relevant signal, fluorescence channels were manually thresholded to generate binary masks. Threshold levels were carefully adjusted to ensure signal detection without over- or under-thresholding. Mean gray values were recorded (arbitrary units, A.U.), and normalized fluorescence intensity values were calculated by dividing signal intensity (e.g. *Lmx1* or *Msx*) by DAPI intensity for each embryo. The per time point averages were plotted over time. The line chart was generated using GraphPad Prism; error bars represent the standard deviation across embryos at each time point. All images were processed using uniform parameters to ensure comparability across time points.

### Single-nucleus quantification of CTCF and statistical analysis

For high-resolution quantification of transcriptional output, corrected total cell fluorescence (CTCF) was calculated for individual nuclei using the formula:

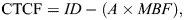
where *ID* is the integrated density, *A* is the area of the ROI and *MBF* is the mean background fluorescence.

Scatter plots displaying the CTCF of *Lmx1* and *Msx* over normalized *y*-coordinates were generated by measuring individual neural nuclei along the dorsal midline using a fixed-size circular ROI with an area of 32.09 px^2^. For each ROI, the mean gray value, integrated density and coordinates were recorded for three embryos per time point (*n*=4). *t*-tests were used to assess differences in CTCF between nuclei at the zippering point and those located anterior or posterior to it. Pairwise differences were assessed using an unpaired, two-tailed Welch's *t*-test (independent samples) implemented with the Scpipy.stats function ttest_ind, followed by Holm-Bonferroni correction for multiple comparisons using statsmodels.stats.multitest. A ridgeline plot displaying posterior-to-anterior *Lmx1* transcription wave during neurulation was generated by recording the *x*- and *y*-coordinates of *Lmx1*-expressing nuclei and then normalizing the coordinates to the bounds of the embryo at respective time points. Graphs were generated using matplotlib.pyplot.

For dorsal midline cell number quantification, we counted the total number of *Lmx1::mnG*- or *Msx::mApp*-labeled nuclei per embryo per condition at 8 h 27 min pf. Each overexpression construct was compared only to its corresponding enhancer-matched *LacZ* control. Statistical significance was assessed via independent Welch's *t*-tests (two-tailed) using the SciPy.stats function ttest_ind.

For EdU incorporation assays, the percentage of EdU^+^ *Lmx1*>*H2B:mApp*-expressing cells was quantified per embryo. Each data point represents one embryo. Statistical significance between *Lmx1*>*LacZ* and *Lmx1*>*Cdkn1b* embryos was tested using the nonparametric Mann–Whitney *U*-test (two-tailed) using the SciPy.stats function mannwhitneyu. Bar graphs display the mean±s.e.m. Figures were generated using seaborn and matplotlib.pyplot.

### Single-cell RNA-seq data analysis

Single-cell RNA-seq data from *Ciona intestinalis* type A (formerly *Ciona robusta*) initial tailbud I embryos (SRA accession numbers SRR9050993 and SRR9050994) were obtained from a previously published study (Cao et al., 2019). Raw reads were aligned to the latest *Ciona* genome assembly (HT) and the updated gene model (KY21) using 10x Genomics Cell Ranger (v2.0.1).

Post-alignment, single-cell data were analyzed using the Scanpy Python library (Wolf et al., 2018). Low-quality cells were filtered out by removing cells in which >20% of total counts were attributed to mitochondrial genes, <200 expressed genes, or a total number of unique molecular identifiers (UMIs) exceeding three standard deviations above the mean. Genes expressed in fewer than three cells were also excluded. Doublets were detected and removed using Scrublet (Wolock et al., 2019). Following filtering, 4526 cells and 15,290 genes remained for downstream analysis.

Gene expression values were normalized to the median total count per cell using scanpy.pp.normalize_total, followed by log-transformation with scanpy.pp.log1p. Highly variable genes were identified using scanpy.pp.highly_variable_genes with the Seurat v3 method (flavor=‘seurat_v3’, top 2000 genes). These genes were scaled to unit variance and centered (values clipped at a maximum of 10) using scanpy.pp.scale.

Principal component analysis (PCA) was performed using the ARPACK solver. To correct for sample-specific batch effects, the top principal components were adjusted using the Harmony algorithm (Korsunsky et al., 2019), and the harmonized representation was stored in X_pca_harmony. A neighborhood graph was computed using scanpy.pp.neighbors on the Harmony-corrected components (*n*_neighbors=30, *n*_pcs=50).

Uniform Manifold Approximation and Projection (UMAP) was then applied for dimensionality reduction and visualization (McInnes et al., 2018) to capture subpopulation heterogeneity. CELF3 (also called ETR-1) was used as a neural marker for visualization and annotation of neural clusters in UMAP projections, based on its established expression in the *Ciona* nerve cord (Yagi and Makabe, 2001; Satou et al., 2001).

## Supplementary Material

10.1242/develop.205064_sup1 Supplementary information

## References

[DEV205064C1] Abitua, P. B., Wagner, E., Navarrete, I. A. and Levine, M. (2012). Identification of a rudimentary neural crest in a non-vertebrate chordate. *Nature* 492, 104-107. 10.1038/nature11589 23135395 PMC4257486

[DEV205064C2] Angelini, T. E., Hannezo, E., Trepat, X., Marquez, M., Fredberg, J. J. and Weitz, D. A. (2011). Glass-like dynamics of collective cell migration. *Proc. Natl. Acad. Sci. USA* 108, 4714-4719. 10.1073/pnas.1010059108 21321233 PMC3064326

[DEV205064C3] Avagliano, L., Massa, V., George, T. M., Qureshy, S., Bulfamante, G. P. and Finnell, R. H. (2019). Overview on neural tube defects: from development to physical characteristics. *Birth Defects Res.* 111, 1455-1467. 10.1002/bdr2.1380 30421543 PMC6511489

[DEV205064C4] Bocanegra-Moreno, L., Singh, A., Hannezo, E., Zagorski, M. and Kicheva, A. (2023). Cell cycle dynamics control fluidity of the developing mouse neuroepithelium. *Nat. Phys.* 19, 1050-1058. 10.1038/s41567-023-01977-w 37456593 PMC10344780

[DEV205064C5] Cao, C., Lemaire, L. A., Wang, W., Yoon, P. H., Choi, Y. A., Parsons, L. R., Matese, J. C., Wang, W., Levine, M. and Chen, K. (2019). Comprehensive single-cell transcriptome lineages of a proto-vertebrate. *Nature* 571, 349-354. 10.1038/s41586-019-1385-y 31292549 PMC6978789

[DEV205064C6] Chizhikov, V. V. and Millen, K. J. (2004). Control of roof plate formation by Lmx1a in the developing spinal cord. *Development* 131, 2693-2705. 10.1242/dev.01139 15148302

[DEV205064C7] Christiaen, L., Wagner, E., Shi, W. and Levine, M. (2009a). Electroporation of transgenic DNAs in the sea squirt Ciona. *Cold Spring Harb. Protoc.* 2009, pdb.prot5345. 10.1101/pdb.prot5345 20150092

[DEV205064C8] Christiaen, L., Wagner, E., Shi, W. and Levine, M. (2009b). Isolation of sea squirt (Ciona) gametes, fertilization, dechorionation, and development. *Cold Spring Harb. Protoc.* 2009, pdb.prot5344. 10.1101/pdb.prot5344 20150091

[DEV205064C9] Clark, E., Peel, A. D. and Akam, M. (2019). Arthropod segmentation. *Development* 146, dev170480. 10.1242/dev.170480 31554626

[DEV205064C10] Dahlberg, C., Auger, H., Dupont, S., Sasakura, Y., Thorndyke, M. and Joly, J.-S. (2009). Refining the Ciona intestinalis model of central nervous system regeneration. *PLoS ONE* 4, e4458. 10.1371/journal.pone.0004458 19212465 PMC2639796

[DEV205064C11] Fujiwara, D., Yoshimoto, H., Sone, H., Harashima, S. and Tamai, Y. (1998). Transcriptional co-regulation of Saccharomyces cerevisiae alcohol acetyltransferase gene, ATF1 and delta-9 fatty acid desaturase gene, OLE1 by unsaturated fatty acids. *Yeast* 14, 711-721. 10.1002/(SICI)1097-0061(19980615)14:8<711::AID-YEA263>3.0.CO;2-8 9675816

[DEV205064C12] Gainous, T. B., Wagner, E. and Levine, M. (2015). Diverse ETS transcription factors mediate FGF signaling in the Ciona anterior neural plate. *Dev. Biol.* 399, 218-225. 10.1016/j.ydbio.2014.12.032 25576927 PMC4851347

[DEV205064C13] German, M. S., Wang, J., Chadwick, R. B. and Rutter, W. J. (1992). Synergistic activation of the insulin gene by a LIM-homeo domain protein and a basic helix-loop-helix protein: building a functional insulin minienhancer complex. *Genes Dev.* 6, 2165-2176. 10.1101/gad.6.11.2165 1358758

[DEV205064C14] Greene, N. D. E. and Copp, A. J. (2014). Neural tube defects. *Annu. Rev. Neurosci.* 37, 221-242. 10.1146/annurev-neuro-062012-170354 25032496 PMC4486472

[DEV205064C15] Gregory, C. and Veeman, M. (2013). 3D-printed microwell arrays for Ciona microinjection and timelapse imaging. *PLoS ONE* 8, e82307. 10.1371/journal.pone.0082307 24324769 PMC3855702

[DEV205064C16] Harris, M. J. and Juriloff, D. M. (2007). Mouse mutants with neural tube closure defects and their role in understanding human neural tube defects. *Birth Defects Res. A Clin. Mol. Teratol.* 79, 187-210. 10.1002/bdra.20333 17177317

[DEV205064C17] Harris, M. J. and Juriloff, D. M. (2010). An update to the list of mouse mutants with neural tube closure defects and advances toward a complete genetic perspective of neural tube closure. *Birth Defects Res. A Clin. Mol. Teratol.* 88, 653-669. 10.1002/bdra.20676 20740593

[DEV205064C18] Hashimoto, H. and Munro, E. (2019). Differential expression of a classic cadherin directs tissue-level contractile asymmetry during neural tube closure. *Dev. Cell* 51, 158-172.e4. 10.1016/j.devcel.2019.10.001 31639367 PMC7458479

[DEV205064C19] Hashimoto, H., Robin, F. B., Sherrard, K. M. and Munro, E. M. (2015). Sequential contraction and exchange of apical junctions drives zippering and neural tube closure in a simple chordate. *Dev. Cell* 32, 241-255. 10.1016/j.devcel.2014.12.017 25625209

[DEV205064C20] Hotta, K., Mitsuhara, K., Takahashi, H., Inaba, K., Oka, K., Gojobori, T. and Ikeo, K. (2007). A web-based interactive developmental table for the ascidian Ciona intestinalis, including 3D real-image embryo reconstructions: I. from fertilized egg to hatching larva. *Dev. Dyn.* 236, 1790-1805. 10.1002/dvdy.21188 17557317

[DEV205064C21] Imai, K. S., Hino, K., Yagi, K., Satoh, N. and Satou, Y. (2004). Gene expression profiles of transcription factors and signaling molecules in the ascidian embryo: towards a comprehensive understanding of gene networks. *Development* 131, 4047-4058. 10.1242/dev.01270 15269171

[DEV205064C22] Imai, K. S., Levine, M., Satoh, N. and Satou, Y. (2006). Regulatory blueprint for a chordate embryo. *Science* 312, 1183-1187. 10.1126/science.1123404 16728634

[DEV205064C23] Irvine, K. D. and Wieschaus, E. (1994). Cell intercalation during Drosophila germband extension and its regulation by pair-rule segmentation genes. *Development* 120, 827-841. 10.1242/dev.120.4.827 7600960

[DEV205064C24] Ishida, T. and Satou, Y. (2024). Ascidian embryonic cells with properties of neural-crest cells and neuromesodermal progenitors of vertebrates. *Nat. Ecol. Evol.* 8, 1154-1164. 10.1038/s41559-024-02387-8 38565680

[DEV205064C25] José-Edwards, D. S., Kerner, P., Kugler, J. E., Deng, W., Jiang, D. and Di Gregorio, A. (2011). The identification of transcription factors expressed in the notochord of Ciona intestinalis adds new potential players to the Brachyury gene regulatory network. *Dev. Dyn.* 240, 1793-1805. 10.1002/dvdy.22656 21594950 PMC3685856

[DEV205064C26] Kancherla, V. (2023). Neural tube defects: a review of global prevalence, causes, and primary prevention. *Childs. Nerv. Syst.* 39, 1703-1710. 10.1007/s00381-023-05910-7 36882610

[DEV205064C27] Khoueiry, P., Rothbächer, U., Ohtsuka, Y., Daian, F., Frangulian, E., Roure, A., Dubchak, I. and Lemaire, P. (2010). A cis-regulatory signature in ascidians and flies, independent of transcription factor binding sites. *Curr. Biol.* 20, 792-802. 10.1016/j.cub.2010.03.063 20434338

[DEV205064C28] Kim, K., Orvis, J. and Stolfi, A. (2022). Pax3/7 regulates neural tube closure and patterning in a non-vertebrate chordate. *Front. Cell Dev. Biol* 10, 999511. 10.3389/fcell.2022.999511 36172287 PMC9511217

[DEV205064C29] Kim, S., Pochitaloff, M., Stooke-Vaughan, G. A. and Campàs, O. (2021). Embryonic tissues as active foams. *Nat. Phys.* 17, 859-866. 10.1038/s41567-021-01215-1 34367313 PMC8336761

[DEV205064C30] Kobayashi, K., Tokuoka, M., Sato, H., Ariyoshi, M., Kawahara, S., Fujiwara, S., Kishimoto, T. and Satou, Y. (2022). Regulators specifying cell fate activate cell cycle regulator genes to determine cell numbers in ascidian larval tissues. *Development* 149, dev201218. 10.1242/dev.201218 36278804

[DEV205064C31] Korsunsky, I., Millard, N., Fan, J., Slowikowski, K., Zhang, F., Wei, K., Baglaenko, Y., Brenner, M., Loh, P.-R. and Raychaudhuri, S. (2019). Fast, sensitive and accurate integration of single-cell data with Harmony. *Nat. Methods* 16, 1289-1296. 10.1038/s41592-019-0619-0 31740819 PMC6884693

[DEV205064C32] Lehr, S., Brückner, D. B., Minchington, T. G., Greunz-Schindler, M., Merrin, J., Hannezo, E. and Kicheva, A. (2025). Self-organized pattern formation in the developing mouse neural tube by a temporal relay of BMP signaling. *Dev. Cell* 60, 567-580.e14. 10.1016/j.devcel.2024.10.024 39603235

[DEV205064C33] Lemaire, L. A., Cao, C., Yoon, P. H., Long, J. and Levine, M. (2021). The hypothalamus predates the origin of vertebrates. *Sci. Adv.* 7, eabf7452. 10.1126/sciadv.abf7452 33910896 PMC8081355

[DEV205064C34] McInnes, L., Healy, J., Saul, N. and Großberger, L. (2018). UMAP: Uniform Manifold Approximation and Projection. *J. Open Source Software* 3, 861. 10.21105/joss.00861

[DEV205064C35] Millonig, J. H., Millen, K. J. and Hatten, M. E. (2000). The mouse dreher gene Lmx1a controls formation of the roof plate in the vertebrate CNS. *Nature* 403, 764-769. 10.1038/35001573 10693804

[DEV205064C36] Mishima, Y., Lindgren, A. G., Chizhikov, V. V., Johnson, R. L. and Millen, K. J. (2009). Overlapping function of Lmx1a and Lmx1b in anterior hindbrain roof plate formation and cerebellar growth. *J. Neurosci.* 29, 11377-11384. 10.1523/JNEUROSCI.0969-09.2009 19741143 PMC2765661

[DEV205064C37] Moon, L. D. and Xiong, F. (2022). Mechanics of neural tube morphogenesis. *Semin. Cell Dev. Biol.* 130, 56-69. 10.1016/j.semcdb.2021.09.009 34561169

[DEV205064C38] Negishi, T., McDougall, A. and Yasuo, H. (2013). Practical tips for imaging ascidian embryos. *Dev. Growth Differ.* 55, 446-453. 10.1111/dgd.12059 23611302

[DEV205064C39] Negrón-Piñeiro, L. J., Wu, Y., Popsuj, S., José-Edwards, D. S., Stolfi, A. and Di Gregorio, A. (2024). Cis-regulatory interfaces reveal the molecular mechanisms underlying the notochord gene regulatory network of Ciona. *Nat. Commun.* 15, 3025. 10.1038/s41467-024-46850-3 38589372 PMC11001920

[DEV205064C40] Nikolopoulou, E., Galea, G. L., Rolo, A., Greene, N. D. E. and Copp, A. J. (2017). Neural tube closure: cellular, molecular and biomechanical mechanisms. *Development* 144, 552-566. 10.1242/dev.145904 28196803 PMC5325323

[DEV205064C41] Nüsslein-Volhard, C. and Wieschaus, E. (1980). Mutations affecting segment number and polarity in drosophila. *Nature* 287, 795-801. 10.1038/287795a0 6776413

[DEV205064C42] Ogura, Y. and Sasakura, Y. (2016). Developmental control of cell-cycle compensation provides a switch for patterned mitosis at the onset of chordate neurulation. *Dev. Cell* 37, 148-161. 10.1016/j.devcel.2016.03.013 27093084

[DEV205064C43] Ogura, Y., Sakaue-Sawano, A., Nakagawa, M., Satoh, N., Miyawaki, A. and Sasakura, Y. (2011). Coordination of mitosis and morphogenesis: role of a prolonged G2 phase during chordate neurulation. *Development* 138, 577-587. 10.1242/dev.053132 21205801

[DEV205064C44] Ramos, C. and Robert, B. (2005). Msh/Msx gene family in neural development. *Trends Genet.* 21, 624-632. 10.1016/j.tig.2005.09.001 16169630

[DEV205064C45] Riddle, R. D., Ensini, M., Nelson, C., Tsuchida, T., Jesseil, T. M. and Tabin, C. (1995). Induction of the LIM homeobox gene Lmx1 by WNT6a establishes dorsoventral pattern in the vertebrate limb. *Cell* 83, 631-640. 10.1016/0092-8674(95)90103-5 7585966

[DEV205064C46] Roure, A. and Darras, S. (2016). Msxb is a core component of the genetic circuitry specifying the dorsal and ventral neurogenic midlines in the ascidian embryo. *Dev. Biol.* 409, 277-287. 10.1016/j.ydbio.2015.11.009 26592100

[DEV205064C47] Sakamoto, R., Banerjee, D. S., Yadav, V., Chen, S., Gardel, M. L., Sykes, C., Banerjee, S. and Murrell, M. P. (2023). Membrane tension induces F-actin reorganization and flow in a biomimetic model cortex. *Commun. Biol.* 6, 325. 10.1038/s42003-023-04684-7 36973388 PMC10043271

[DEV205064C48] Sasakura, Y., Mita, K., Ogura, Y. and Horie, T. (2012). Ascidians as excellent chordate models for studying the development of the nervous system during embryogenesis and metamorphosis. *Dev. Growth Differ.* 54, 420-437. 10.1111/j.1440-169X.2012.01343.x 22524611

[DEV205064C49] Satou, Y., Takatori, N., Yamada, L., Mochizuki, Y., Hamaguchi, M., Ishikawa, H., Chiba, S., Imai, K., Kano, S., Murakami, S. D. et al. (2001). Gene expression profiles in *Ciona intestinalis* tailbud embryos. *Development* 128, 2893-2904. 10.1242/dev.128.15.2893 11532913

[DEV205064C50] Schindelin, J., Arganda-Carreras, I., Frise, E., Kaynig, V., Longair, M., Pietzsch, T., Preibisch, S., Rueden, C., Saalfeld, S., Schmid, B. et al. (2012). Fiji: an open-source platform for biological-image analysis. *Nat. Methods* 9, 676-682. 10.1038/nmeth.2019 22743772 PMC3855844

[DEV205064C51] Todorov, L. G., Oonuma, K., Kusakabe, T. G., Levine, M. S. and Lemaire, L. A. (2024). Neural crest lineage in the protovertebrate model Ciona. *Nature* 635, 912-916. 10.1038/s41586-024-08111-7 39443803 PMC12906288

[DEV205064C52] Traag, V. A., Waltman, L. and Van Eck, N. J. (2019). From Louvain to Leiden: guaranteeing well-connected communities. *Sci. Rep.* 9, 5233. 10.1038/s41598-019-41695-z 30914743 PMC6435756

[DEV205064C53] Treen, N., Chavarria, E., Weaver, C. J., Brangwynne, C. P. and Levine, M. (2023a). An FGF timer for zygotic genome activation. *Genes Dev.* 37, 80-85. 10.1101/gad.350164.122 36801820 PMC10069452

[DEV205064C54] Treen, N., Konishi, S., Nishida, H., Onuma, T. A. and Sasakura, Y. (2023b). Zic-r.b controls cell numbers in ciona embryos by activating CDKN1B. *Dev. Biol.* 498, 26-34. 10.1016/j.ydbio.2023.03.005 36965841

[DEV205064C55] Wolf, F. A., Angerer, P. and Theis, F. J. (2018). SCANPY: large-scale single-cell gene expression data analysis. *Genome Biol.* 19, 15. 10.1186/s13059-017-1382-0 29409532 PMC5802054

[DEV205064C56] Wolock, S. L., Lopez, R. and Klein, A. M. (2019). Scrublet: computational identification of cell doublets in single-cell transcriptomic data. *Cell Syst.* 8, 281-291.e9. 10.1016/j.cels.2018.11.005 30954476 PMC6625319

[DEV205064C57] Yagi, K. and Makabe, K. W. (2001). Isolation of an early neural marker gene abundantly expressed in the nervous system of the ascidian, *Halocynthia roretzi*. *Dev. Genes Evol.* 211, 49-53. 10.1007/s004270000118 11277406

[DEV205064C58] Yan, C. H., Levesque, M., Claxton, S., Johnson, R. L. and Ang, S.-L. (2011). Lmx1a and Lmx1b function cooperatively to regulate proliferation, specification, and differentiation of midbrain dopaminergic progenitors. *J. Neurosci.* 31, 12413-12425. 10.1523/JNEUROSCI.1077-11.2011 21880902 PMC6703256

[DEV205064C59] Zallen, J. A. and Wieschaus, E. (2004). Patterned gene expression directs bipolar planar polarity in Drosophila. *Dev. Cell* 6, 343-355. 10.1016/S1534-5807(04)00060-7 15030758

[DEV205064C60] Zhao, J., Perkins, M. L., Norstad, M. and Garcia, H. G. (2023). A bistable autoregulatory module in the developing embryo commits cells to binary expression fates. *Curr. Biol.* 33, 2851-2864.e11. 10.1016/j.cub.2023.06.060 37453424 PMC10428078

