# Supplementary material for: Pair-rule-like transcription patterns during neural tube closure in a proto-vertebrate
Source: Development. 2025 Dec 15;152(24):dev205064. doi: 10.1242/dev.205064 (PMC12752508; doi:10.1242/dev.205064)
Supplement: Supplementary information [file develop-152-205064-s1.pdf]

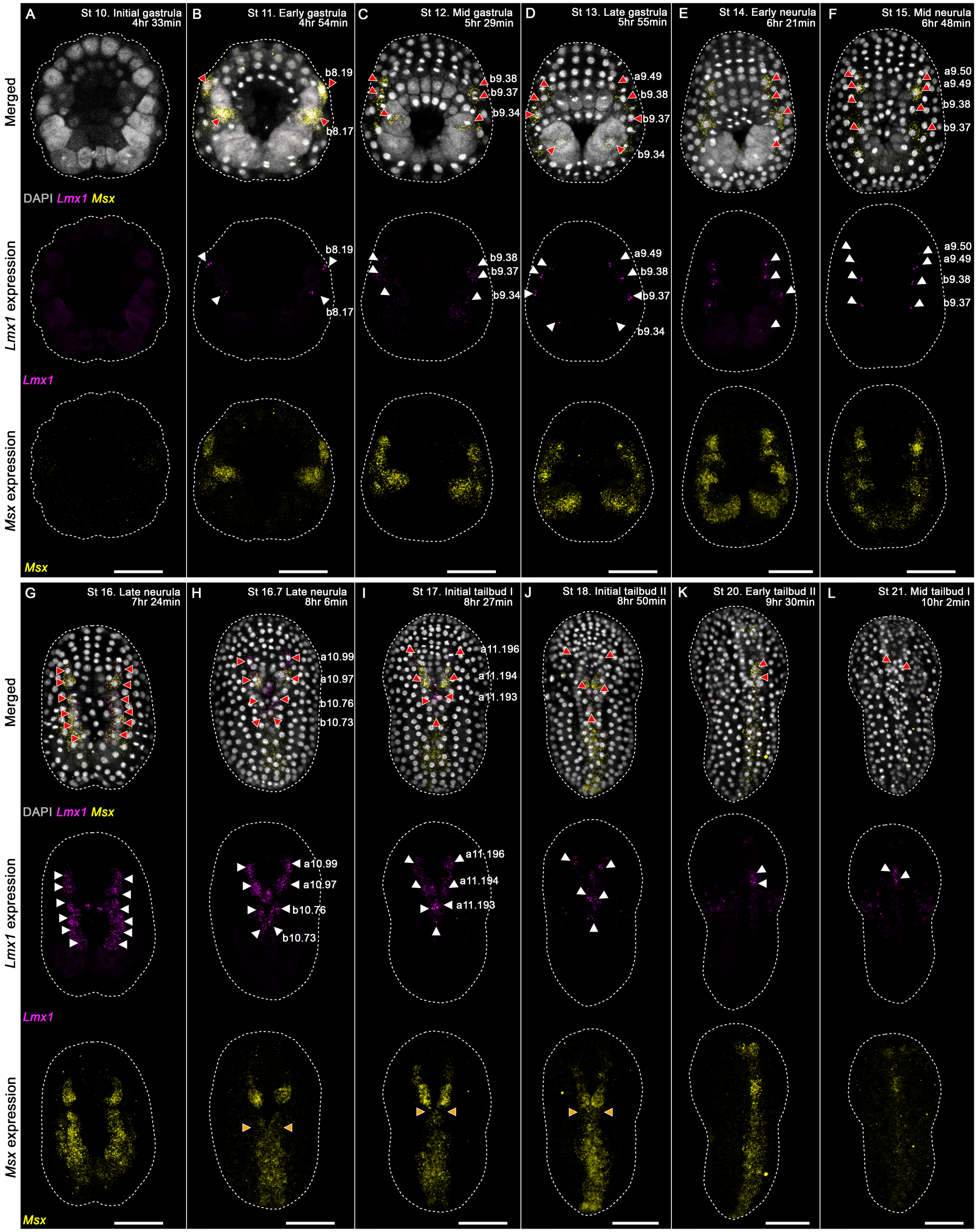

Fig. S1. *Lmx1* and *Msx* expression patterns throughout gastrulation and neurulation. (A-L) Expression was examined throughout neurulation (~4.5-10 hfp; samples collected every ~20 minutes; 12 time points) via HCR *in situ* hybridization. The photographs are maximum-intensity projections of Z-projected image stacks overlaid in pseudocolor with HCR signals for *Lmx1* probe (magenta) and *Msx* probe (yellow). Nuclei were stained with DAPI (gray). Arrowheads (red/white) indicate *Lmx1* expression in descendants of neural plate border cells and neural plate cells, and (orange) *Msx* downregulation at zippering point. Brightness and contrast were adjusted linearly. Numbers of embryos examined n = 15 per time point over n = 5 experiments. Scale bars: 50 µm.

Development • Supplementary information

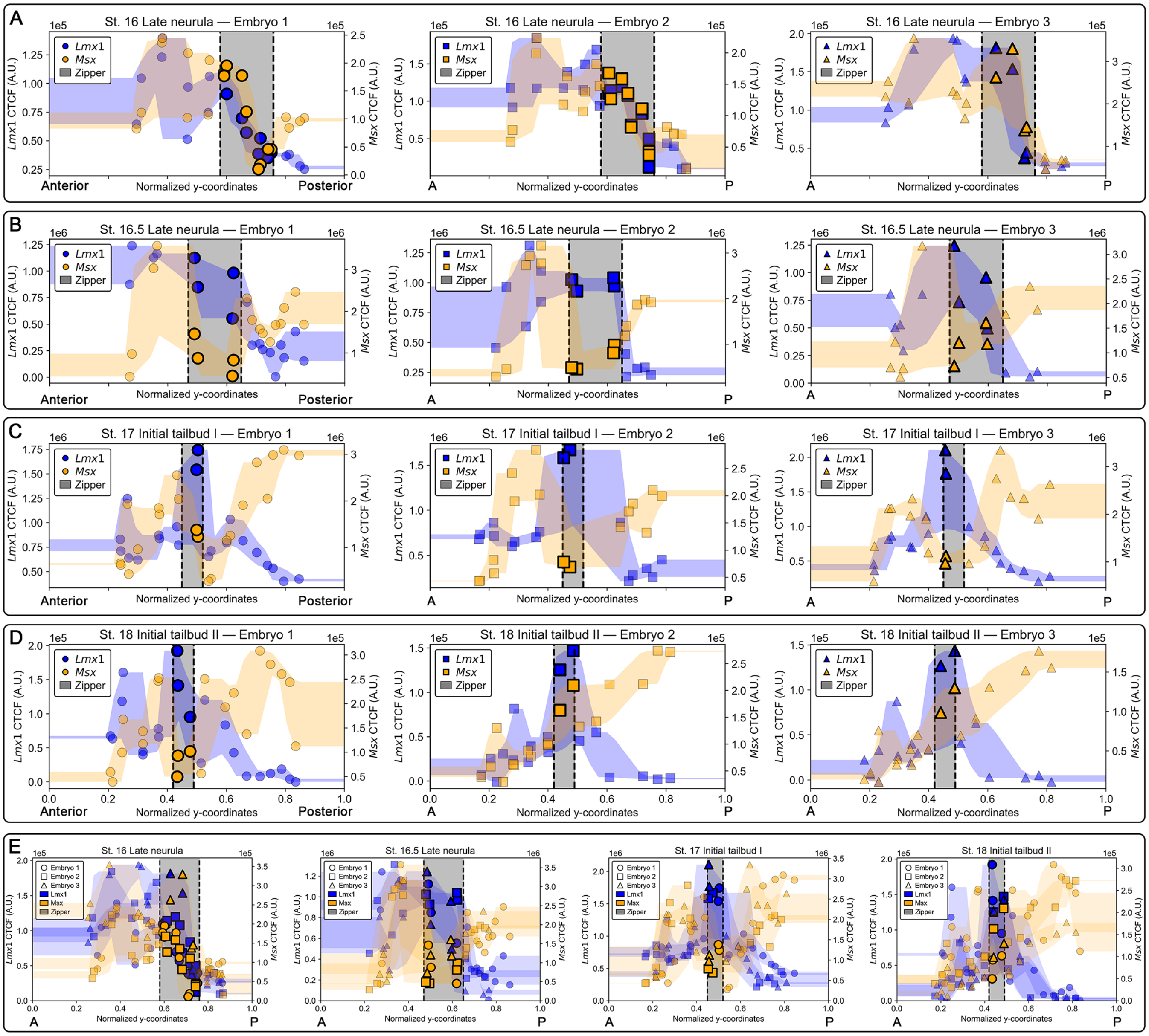

Fig. S2. ***Lmx1*** **expression increases while** ***Msx*** **is selectively downregulated at the zippering point during neural tube closure.**

(A–D) Corrected total cell fluorescence (CTCF) for *Lmx1* (dark blue) and *Msx* (orange) HCR *in situ* hybridization signals in dorsal midline cells across four developmental stages of NTC, plotted by each cell's normalized y-coordinate (relative to the embryo bounds). Each point represents a single nucleus, and shaded regions denote the local range across neighboring points. Data are shown for n = 3 embryos per time point (n = 4), with marker shapes indicating individual embryos. During the Late Neurula stage (A), *Lmx1* and *Msx* are broadly co-expressed. As zippering initiates and progresses (B–C), *Lmx1* becomes locally enriched at the zippering point (gray region between dashed lines), while *Msx* becomes selectively downregulated in the same domain. (E) Aggregate plots from (A–D) illustrate consistency and embryo-to-embryo variability.

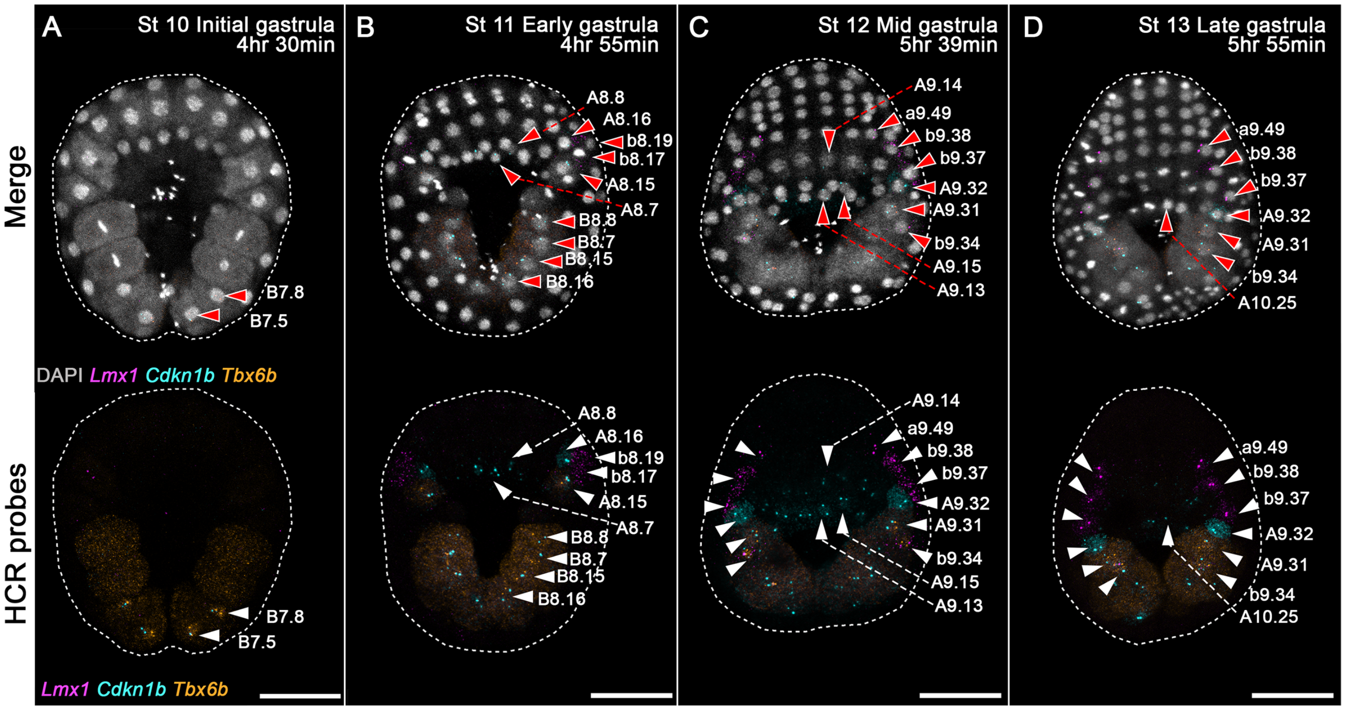

Fig. S3. Lmx1, Cdkn1b, and Tbx6-b expression patterns during gastrulation.
(A-D) Expression was examined throughout gastrulation (∼4.5-6 hfp; samples collected every ∼30 minutes; four time points) via HCR *in situ* hybridization. The photographs are maximum-intensity projections of Z-projected image stacks overlaid in pseudocolor with HCR signals for *Lmx1* probe (magenta), Tbx6-b probe (orange), and *Cdkn1b* probe (cyan). Nuclei were stained with DAPI (gray). Developmental stages are indicated in the photographs. Arrowheads (red/white) indicate cells that express *Lmx1* and/or *Cdkn1b* and contribute to the future CNS. Brightness and contrast were adjusted linearly. Numbers of embryos examined n = 15 per time point over n = 3 experiments. Scale bars: 50 µm.

Development • Supplementary information

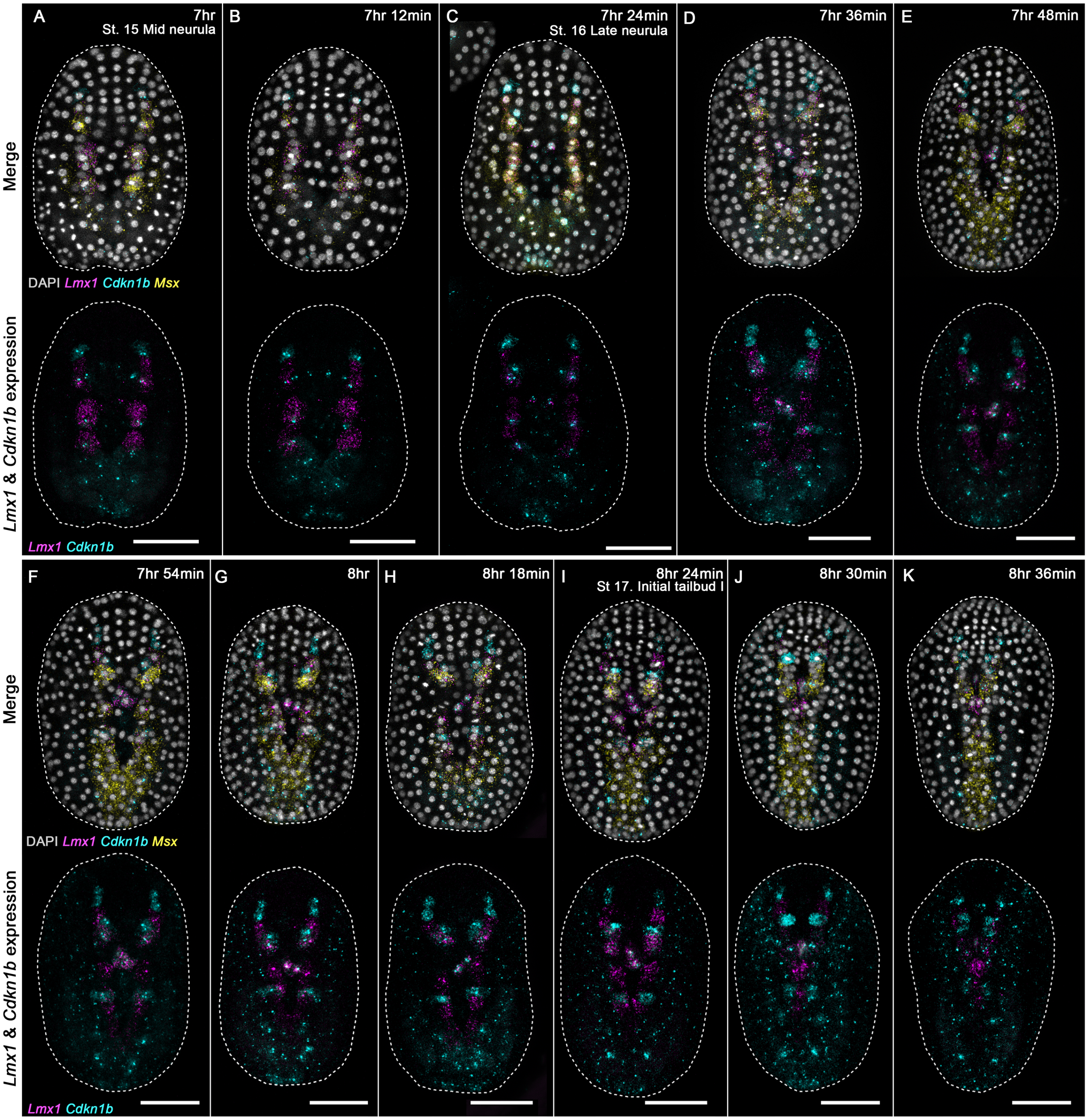

Fig. S4. *Lmx1*, *Msx*, and *Cdkn1b* expression patterns during neurulation. (A-K) Expression was examined throughout neural tube closure (~7-8.5 hfp; samples collected every ~6 minutes; 13 time points) via HCR *in situ* hybridization. The photographs are maximum-intensity projections of Z-projected image stacks overlaid in pseudocolor with HCR signals for *Lmx1* probe (magenta), *Msx* probe (yellow), and *Cdkn1b* probe (cyan). Nuclei were stained with DAPI (gray). Developmental stages are indicated in the photographs. Brightness and contrast were adjusted linearly. Numbers of embryos examined n = 15 per time point over n = 3 experiments. Scale bars: 50 µm.

Table S1. Gene identifiers.

Gene name	KY21 Model	KH2013 Model	Regulatory sequence	Human homolog	Synonyms	ISH	Reporter
Lmx1	KY21.Chr9.606	KH.C9.616	KY21.Chr9:4348500... 4350529	LMX1B	HGNC:6654, LIM homeobox transcription factor 1 beta	Fig. 1, 2, 4, S1, S3, S4	Fig. 2, 3
Cdkn1b	KY21.Chr2.18	KH.S643.6		CDKN1B	HGNC:1785, cyclin dependent kinase inhibitor 1B	Fig. 4, S3, S4	
Msx	KY21.Chr2.1031	KH.C2.957	KY21.Chr2:6131140... 6133586	MSX2	HGNC:7392, msh homeobox 2	Fig. 1, 2, 4, S1, S4	Fig. 2, 3, Movie S1
Sox1/2/3	KY21.Chr1.254	KH.C1.99		SOX2	HGNC:11195, SRY-box transcription factor 2		Movie S1
cdc25a	KY21.Chr5.730	KH.C5.12		CDC25A	HGNC:1725, cell division cycle 25A		
Cdk1	KY21.Chr12.455	KH.C12.372		CDK1	HGNC:1722, cyclin dependent kinase 1		
Cdk2/3	KY21.Chr1.1716			CDK2	HGNC:1771, cyclin dependent kinase 2		
TOP2A	KY21.Chr2.871	KH.C2.374		TOP2A	HGNC:11989, DNA topoisomerase II alpha		
Celf3.a	KY21.Chr6.58	KH.C6.128		CELF3	HGNC:11967, CUGBP Elav-like family member 3		
ZCCHC24	KY21.Chr14.222	KH.C14.310		ZCCHC24	HGNC:26911, zinc finger CCHC-type containing 24		
SLC35F6	KY21.Chr4.712	KH.C4.90		SLC35F6	HGNC:26055, solute carrier family 35 member F6		
Wnt7	KY21.Chr8.1169	KH.C8.843		WNT7B	HGNC:12787, Wnt family member 7B		
Wnt9	KY21.Chr9.626			WNT9B	HGNC:12779, Wnt family member 9B		
Admp	KY21.Chr2.381	KH.C2.421		BMP3	HGNC:1070, bone morphogenetic protein 3		
Nog (noggin)	KY21.Chr12.737	KH.C12.562		NOG	HGNC:7866, noggin		
(chordin)	KY21.Chr6.382	KH.C6.145		CHRD	HGNC:1949, chordin		
Zic-r.a	KY21.Chr1.1337	KH.C1.727		ZIC3	HGNC:12874, Zic family member 3		
Smad1/5/9	KY21.Chr2.623	KH.C2.573		SMAD1	HGNC:6767, SMAD family member 1		
Fgf3/7/10/22	KY21.Chr11.1298	KH.S406.19		FGF7	HGNC:3685, fibroblast growth factor 7		
YAP1	KY21.Chr7.860	KH.C7.459		YAP1	HGNC:16262, Yes1 associated transcriptional regulator		
Cralbp	KY21.Chr11.800	KH.C11.439		RLBP1	HGNC:10024, retinaldehyde binding protein 1)		
Tbx6-r.b	KY21.Chr11.467	KH.S654.3		TBX6	HGNC:11605, T-box transcription factor 6	Fig. S3	

Table S2. HCR Probe cDNA sequence identifiers.

Gene name	KY21 Model	KH2012 Model	cDNA sequence	Probe	GhostHT URL	Vendor
Lmx1	KY21.Chr9.606	KH.C9.616	>KY21.Chr9.606.v1.SL2-1	B1	http://ghost.zool.kyoto-u.ac.jp/cgi-bin/fordetailky21.cgi?name=KY21.Chr9.606.v1.SL2-1	Molecular Instruments
Cdkn1b	KY21.Chr2.18	KH.S643.6	>KY21.Chr2.18.v1.SL1-1	B3	http://ghost.zool.kyoto-u.ac.jp/cgi-bin/fordetailky21.cgi?name=KY21.Chr2.18.v1.SL1-1	Molecular Instruments
Msx	KY21.Chr2.1031	KH.C2.957	>KY21.Chr2.1031.v1.nonSL5-1	B5	http://ghost.zool.kyoto-u.ac.jp/cgi-bin/fordetailky21.cgi?name=KY21.Chr2.1031.v1.nonSL5-1	Molecular Instruments

Development • Supplementary information

Table S3. Primers for molecular cloning.

Construct/gene name	Backbone (vector)	Insert (fragment)	Aseembly method	Primer name	Primer sequence	Regulatory / coding sequence
Lmx1>GFP	PCR amplified Lmx1> backbone with 2031 bp Lmx1 regulatory sequence from plasmid Lmx1>H2B::mnG	PCR amplified GFP 717 bp CDS from TRE3G-GFP plasmid gifted by Dr. Charles Ettensohn	Gibson assembly	Lmx1>GFP Frag fwd	cgaattcttacagttatttgtatagttcatccatgccatgtgtaatccc	KH2012.C9:4348500-4350529
				Lmx1>GFP Frag rev	TTTTAGTTCGgccatgagtaaaggagaagaacttttcactgg	
				Lmx1>GFP Vector fwd	ctcctttactcatggcCGAACTAAAATAATTATTCTGCATCGAG	*GFP CDS*
				Lmx1>GFP Vector rev	actatacaaataactgtaagaattcgctgagcgcc	
Msx>Lmx1	PCR amplified Msx> backbone with 2441 bp Msx regulatory sequence from plasmid Msx>H2B::mApp	PCR amplified Lmx1 1875 bp CDS	Gibson assembly	Msx>Lmx1 Frag fwd	aataatacccgtatcactttCGAACTAAAATAATTATTCTGCATCG	KH2012.C2:6131140-6133586
				Msx>Lmx1 Frag rev	cattcaatcctctgATGCTGCGTTCTTCTAACG	
				Msx>Lmx1 Vector fwd	AAGAACGCAGCATcagaggattgaatgcgatcg	KY21.Chr9.606
				Msx>Lmx1 Vector rev	AGAATAATTATTTTAGTTCGaaagtgatacgggtattatttttgc	
Lmx1>Cdkn1b	PCR amplified Lmx1> backbone with 2031 bp Lmx1 regulatory sequence from plasmid Lmx1>H2B::mnG	PCR amplified Cdkn1b 915 bp CDS from plasmid Mesp>CkiB, gifted by Dr. Nicholas Treen	Gibson assembly	Lmx1>Cdkn1.b Frag fwd	ttatcttagtttttcacaggtgtACATATTATTTAAACAATTACTATAAAATGGTTCCCC	KH2012.C9:4348500-4350529
				Lmx1>Cdkn1.b Frag rev	ctgcaataaacaagtCCGAGTTTGTCAG	
				Lmx1>Cdkn1.b Vector fwd	AGTAATTGTTTAAATAATATGTacacctgtgaaaaactaagataattact	KY21.Chr2.18
				Lmx1>Cdkn1.b Vector rev	TTCTGACAAACTCGGacttgtttattgcag	
Mxsb1.5kb>PH::mnG			NotI and AscI restriction enzymes (New England Biolabs) followed by ligation (T4 DNA ligase, Promega)	Msx_Rv	CTACgcggccgcAGAGGATTGAATGCGATCGGATTCG	KH2012.C2:6131140-6133585
				Msx-1.5kb_Fw	CTACggcgcgccaagtccatgcagaccgatgtctattta	
			SpeI restriction enzyme (New England Biolabs)	Degron_Fw	GGTAGTGAGATGAGTGATTCTGATCCAGTAAGACACGAGAGCGCTCCAACATC	Degron degratation signal
				Degron_Rv	GTTTGGAAGTTTTATTTCCATTTTAACTAGTTACTGGATCAGAATCACTCATCTC	
Lmx1 CDS (for Lmx1>Lmx1::VP64 construct)				Lmx1-NotI_Fw	CTACGCGGCCGCAACCATGCTGCGTTCTTCTAACGACGAAA	KY21.Chr9.606
				Lmx1-NaeI_Rv	CTACGCCGGCCGAACTAAAATAATTATTCTGCATCG	

Development • Supplementary information

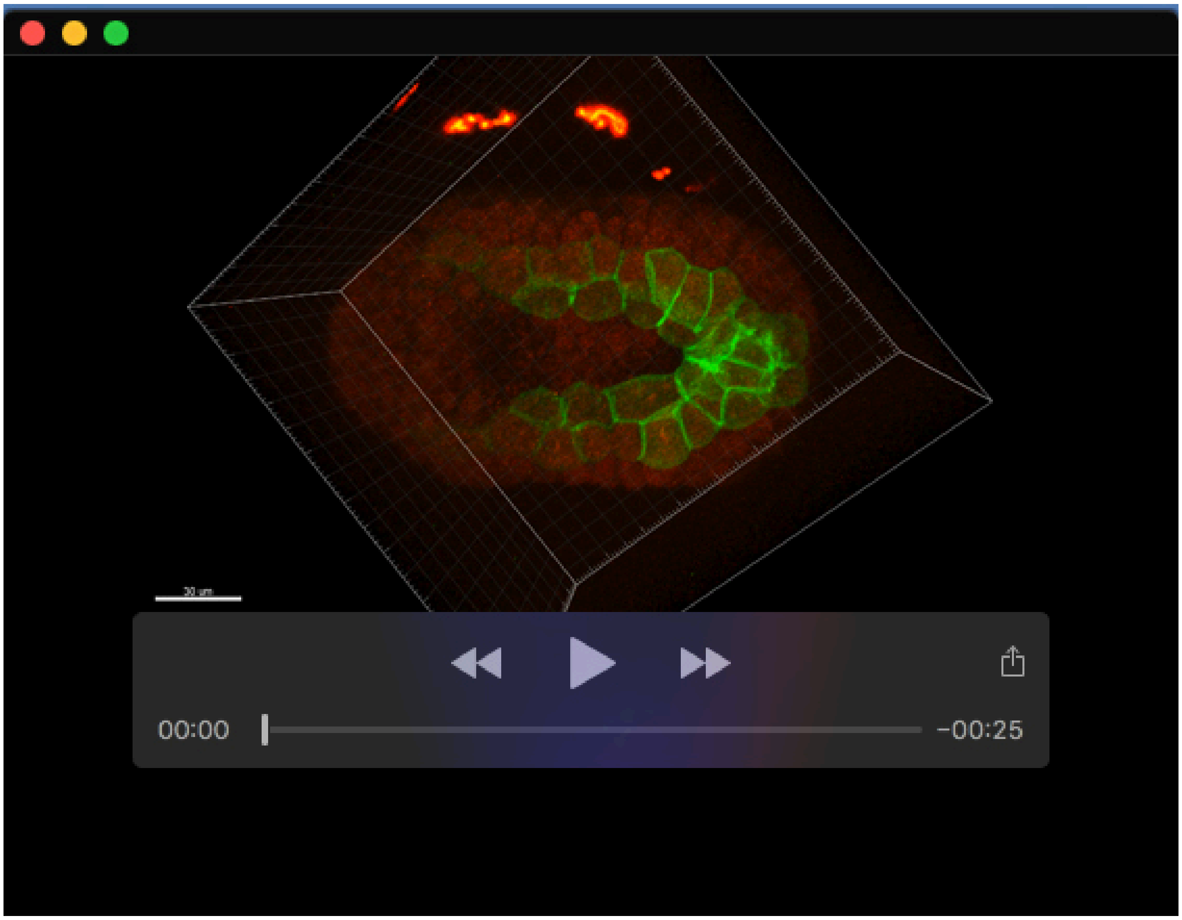

Movie 1. Two-photon live imaging of neural tube closure in *Ciona*. Embryo co-electroporated with *Msxb1.5kb>PH::mnG* (membrane marker; green) and *Sox1/2/3>H2B::mApp* (nuclear marker; red). Time-lapse acquisition began at the early neurula stage (st. 14) and continued until the early tailbud stage (st. 19). The movie shows the posterior-to-anterior progression of the zipper during neural tube closure.
